# CSN6 Promotes Pancreatic Cancer Progression and Gemcitabine Resistance via Antagonizing DCAF1‐Mediated Ubiquitination of NPM1

**DOI:** 10.1002/advs.202510210

**Published:** 2025-10-20

**Authors:** Yijing Zhang, Han Gao, Aiwen Tang, Haiwen Lyu, Zongmin Fan, Jiahui Guo, Yuzhi Wang, Hairong Yi, Qihao Pan, Haidan Luo, Baifu Qin, Boyu Zhang, Xiangqi Meng, Qingxin Liu, Mong‐Hong Lee

**Affiliations:** ^1^ Guangdong Provincial Key laboratory of Colorectal and Pelvic Floor Diseases The Sixth Affiliated Hospital Sun Yat‐sen University Guangzhou 510655 China; ^2^ Guangdong Institute of Gastroenterology Guangzhou 510655 China; ^3^ Department of Oncology The Sixth Affiliated Hospital Sun Yat‐sen University Guangzhou 510655 China; ^4^ Biomedical Innovation Center The Sixth Affiliated Hospital Sun Yat‐sen University Guangzhou 510655 China; ^5^ Institute of Molecular and Medical Virology School of Medicine Jinan University Guangzhou Guangdong Province 510632 China

**Keywords:** CSN6, DCAF1, gemcitabine resistance, NPM1, PDAC

## Abstract

Pancreatic ductal adenocarcinoma (PDAC) is a fatal cancer with poor prognosis. COP9 signalosome subunit 6 (CSN6), a key regulator of different E3 ubiquitin ligases, plays oncogenic roles in various cancers. However, its function in PDAC remains elusive. Here, this demonstrates that human PDAC tumors expressing high levels of CSN6 present with poor prognosis and gemcitabine resistance. Conditional knockout (KO) of CSN6 hinders tumor formation in a KPP spontaneous PDAC mouse model. Proteomic analysis indicates that CSN6 promotes ribosome biogenesis by activating rDNA transcription and protein synthesis. Mechanistically, CSN6 antagonizes DDB1‐CUL4 associated factor 1 (DCAF1)‐mediated ubiquitination of Nucleophosmin (NPM1), thereby promoting NPM1‐orchestrated ribosome biogenesis. In line with CSN6‐mediated gemcitabine resistance, CSN6‐NPM1 axis enhances ribosome biogenesis, thereby promoting translation of gemcitabine resistance genes, including Cytidine deaminase (CDA), Ribonucleotide reductase subunit M1/2 (RRM1/2). Significantly, combining gemcitabine with NPM1 inhibitor NSC348884 synergistically suppresses CSN6‐high pancreatic cancer xenografts. Clinically, CSN6 expression positively correlates with NPM1 in PDAC tissues, and their concurrent high expression is significantly associated with poor clinical outcomes. This study characterizes CSN6 as an oncogenic protein that promotes NPM1 stabilization by interacting with DCAF1, thereby enhancing ribosome biogenesis and cellular resistance to gemcitabine in PDAC. NPM1 may serve as a therapeutic target for CSN6 high PDAC that exhibits gemcitabine drug resistance.

## Graphical abstract

COP9 signalosome subunit 6 (CSN6) promotes the auto‐ubiquitination and degradation of DDB1‐CUL4 associated factor 1 (DCAF1), thereby antagonizing DCAF1‐mediated ubiquitination of Nucleophosmin (NPM1). This stabilization of NPM1 enhances the ribosome biogenesis process and the translation of specific gemcitabine‐resistance proteins, ultimately driving tumor progression and chemoresistance. Conversely, pharmacological inhibition of the CSN6‐NPM1 axis suppresses tumor growth and alleviates gemcitabine resistance.

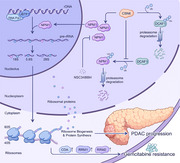

## 1 Introduction

Pancreatic ductal adenocarcinoma (PDAC) is recognized as one of the most lethal and highly aggressive cancers with limited treatment options.^[^ ^1^ ^]^ Gemcitabine, a nucleoside analog, has been applied in the treatment of pancreatic ductal adenocarcinoma.^[^ ^2^, ^3^ ^]^ However, intrinsic or acquired resistance compromises the therapeutic potential of gemcitabine and is the major impediment to achieving expected clinical outcomes.^[^ ^4^ ^]^ Comprehensive genetic and proteomic characterization of PDAC holds promise for developing more effective treatment strategies.^[^ ^5^ ^]^ Also, to propose new treatment options for the large majority of advanced PDAC patients who will face gemcitabine resistance, it has become mandatory to better understand the mechanisms of acquired gemcitabine resistance.

The constitutive photomorphogenesis 9 (COP9) signalosome (CSN) is a highly conserved protein complex present from plants to humans. It functioning as a key regulator in ubiquitin‐mediated protein degradation pathway and participates in diverse biological processes, including signal transduction, cell cycle progression, DNA damage response, and tumorigenesis.^[^ ^6^, ^7^ ^]^ COP9 signalosome subunit 6 (CSN6) is one of the CSN subunits closely involved in the development of multiple cancer types, such as breast cancer, colorectal cancer, cervical cancer, and liver cancer.^[^ ^8^, ^9^, ^10^, ^11^ ^]^ CSN6 engages in diverse biological pathways to regulate proteins such as P53, c‐MYC, β‐catenin, and metabolism‐related proteins such as FASN and PHGDH during tumorigenesis in different cancer types.^[^ ^12^, ^13^ ^]^ Although CSN6 is related to PDAC,^[^ ^14^ ^]^ the molecular mechanism underlying CSN6 deregulation in PDAC remains unclear.

NPM1 (also known as nucleophosmin, B23, NO38, NPM), a nucleocytoplasmic shuttling protein predominantly localized in the nucleolar granular region, is implicated in several cellular functions such as chromatin remodeling, genomic stability, cell cycle, and ribosome biogenesis.^[^ ^15^, ^16^, ^17^, ^18^ ^]^ Emerging evidence suggests that dysregulated ribosome biogenesis is responsible for numerous human diseases including malignant tumor and drug resistance.^[^ ^19^, ^20^ ^]^ Although NPM1 is a key regulator of ribosome biogenesis via facilitating rDNA transcription, pre‐RNA processing, and ribosomal protein maturation,^[^ ^21^ ^]^ its potential role in gemcitabine chemoresistance has not been characterized. Furthermore, while NPM1 activity is known to be modulated by phosphorylation, SUMOylation, and oligomerization, the molecular implications of NPM1 ubiquitination remain elusive.^[^ ^22^ ^]^

In this study, we demonstrate that CSN6 contributes to PDAC progression both in vivo and in vitro. CSN6 expression was significantly elevated in PDAC tumors compared with adjacent normal tissues. Knockdown (KD) or KO of CSN6 attenuated the growth of PDAC cell lines and suppressed tumor formation in a *Kras^G12D^; Tp53^R172H^; Ptf1a‐Cre^ERT2^* (KPP) mouse model. Proteomic analysis revealed that CSN6 participates in ribosome biogenesis and protein synthesis process. Further investigation revealed that CSN6 regulates ribosome biogenesis by modulating the ubiquitination status of NPM1 in a Cullin4‐DCAF1‐dependent manner. Tissue microarray (TMA) analysis demonstrated co‐expression of CSN6 and NPM1 in PDAC specimens, and their occurrent high expression predicted poor patient prognosis. In PDAC cell models of acquired gemcitabine resistance, CSN6 was elevated and promoted cell survival and gemcitabine tolerance. Conversely, CSN6 KD enhanced gemcitabine sensitivity. Mechanistically, CSN6‐mediated NPM1 stabilization facilitated pre‐rRNA synthesis and ribosome maturation, thus enhancing tumorigenesis as well as gemcitabine resistance‐associated proteins’ translation. Thus, CSN6 overexpression (OE) drives gemcitabine resistance by augmenting protein synthesis via NPM1 stabilization, revealing a targetable vulnerability to improve gemcitabine response in PDAC. Accordingly, the NPM1 inhibitor NSC348884 significantly potentiated the antitumor efficacy of gemcitabine in PDAC models. In conclusion, our findings demonstrate that activation of CSN6‐DCAF1‐NPM1 axis is involved in gemcitabine resistance of PDAC cells and can be targeted for advanced PDAC with drug resistance.

## 2 Results

### 2.1 CSN6 is Overexpressed in PDAC, Correlates with Poor Survival, and Promotes Tumorigenesis

**Figure 1:**
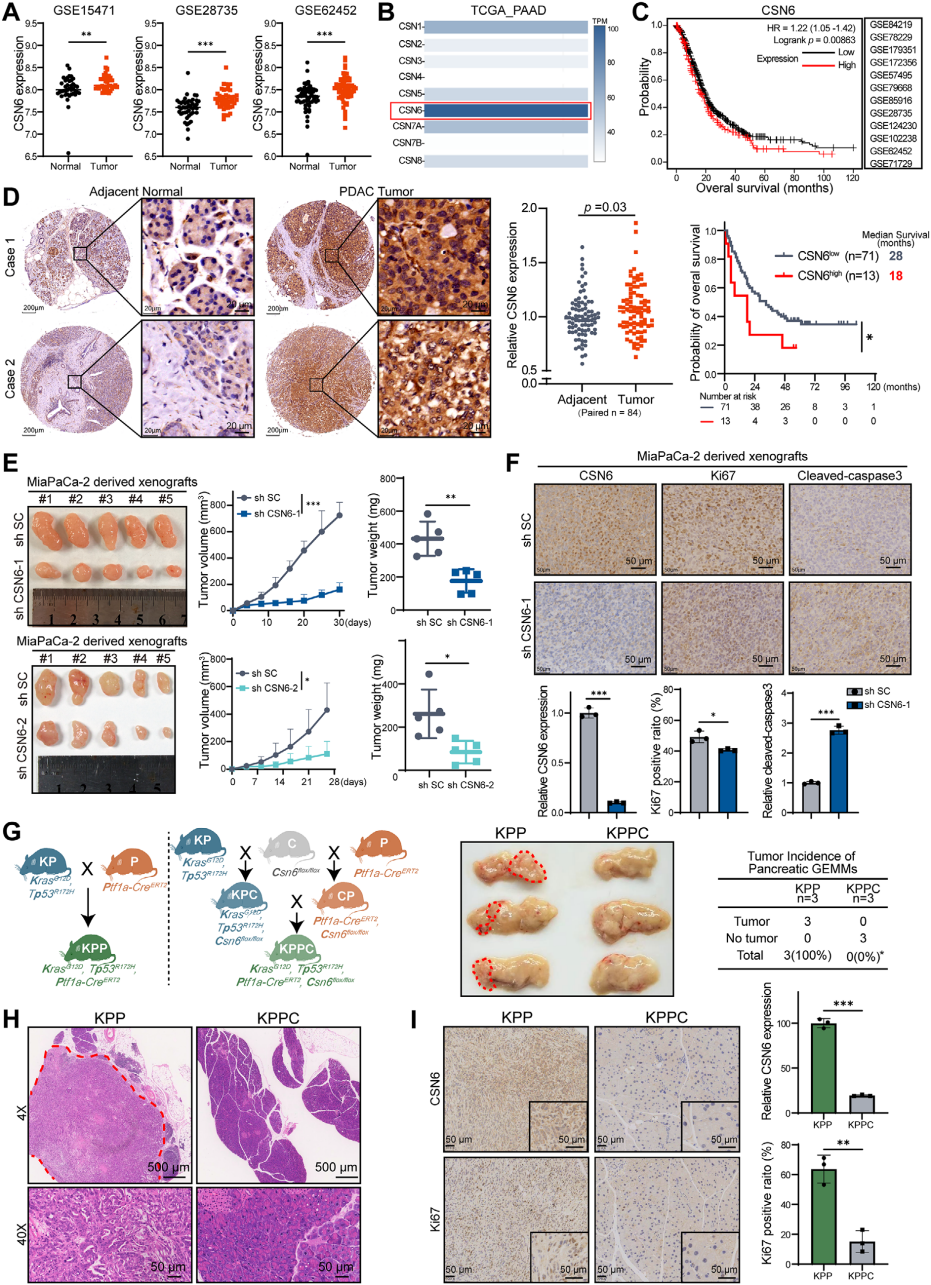
CSN6 is overexpressed in pancreatic ductal adenocarcinoma (PDAC) and drives tumorigenesis. A) CSN6 expression in PDAC versus normal pancreatic tissue across three cohorts (GSE15471, GSE28735, and GSE62452). B) TPM (transcripts per million) heatmap of COP9 signalosome subunits in PDAC. C) Kaplan–Meier survival analysis of patients stratified by CSN6 expression levels across 12 PDAC datasets. D) Representative immunohistochemical (IHC) images of CSN6 in human PDAC tumors and matched adjacent normal tissues (left). Comparison of relative CSN6 expression (H‐score) between tumor and adjacent normal tissues (*n* = 84 paired samples) (middle). Kaplan–Meier analysis of overall survival stratified by CSN6 expression, with the cutoff determined by receiver operating characteristic (ROC) curve analysis (right). E) Subcutaneous xenograft tumors derived from MiaPaCa‐2 cells stably expressing control shRNA (shSC) or shCSN6 (*n* = 5 mice per group, left). Tumor growth curve (middle) and endpoint weights (right) are shown. F) Representative IHC staining for CSN6, Ki67, and cleaved‐caspase3 in xenograft tumors (top). Quantification of the staining intensity using ImageJ (bottom). G) Breeding scheme for generating KPP (*Kras^G12D^, Trp53^R172H^, Ptf1a‐Cre^ERT2^*) and KPPC (*Kras^G12D^, Trp53^R172H^, Ptf1a‐Cre^ERT2^, Csn6^flox/flox^*) mice (left). Macroscopic morphology of pancreas from KPP (*n* = 3) versus KPPC (*n* = 3) mice, with tumor areas outlined by dashed lines (middle). Quantification of tumor incidence (right). H) Representative H&E‐stained sections of KPP pancreas with tumor boundaries indicated. I) IHC staining for CSN6 and Ki67 in pancreatic tissues from KPP (tumor) and KPPC (normal) mice (left). Quantification by ImageJ (right). Data are presented as the mean ± SD. Statistical analyses were performed using Student's *t*‐test (A, D‐middle, E‐right, F, I), log‐rank test (C, D‐right), two‐way ANOVA (E‐middle), or one‐tailed Fisher's exact test (G‐right). Significance is denoted as ^*^ *p* < 0.05, ^**^ *p* < 0.01, ^***^ *p* < 0.001.

Although CSN6 is overexpressed in various types of cancer, its role in PDAC—a highly aggressive malignancy with poor prognosis—remains largely unknown. To investigate the function of CSN6 in PDAC, we analyzed several PDAC gene expression datasets (GSE15471, GSE28735, and GSE62452) and found that CSN6 mRNA level was significantly upregulated in tumor tissues compared to normal samples (**Figure** **1**A). Among all COP9 signalosome subunits, CSN6 exhibited one of the highest expression levels in PDAC based on The Cancer Genome Atlas (TCGA) data (Figure 1B and Figure S1A, Supporting Information). Integrated bioinformatics analysis across 12 independent datasets revealed that high CSN6 expression correlated with poor clinical outcome (Figure 1C). Consistent with these findings, our immunohistochemical (IHC) analysis of a tissue microarray (TMA) containing paired PDAC and normal tissues confirmed a significant elevation of CSN6 protein in tumors (Figure 1D). Kaplan–Meier survival analysis further indicated that high CSN6 levels were associated with reduced overall survival in PDAC patients (Figure 1D). These data indicate that CSN6 is significantly overexpressed in PDAC and may serve as a prognostic marker.

To examine the oncogenic role of CSN6 in PDAC, we performed transient transfection and CSN6‐targeting shRNAs in PDAC cells and conducted cell proliferation assays. The in vitro results demonstrated that CSN6 promotes the growth of PDAC cells, whereas its KD significantly suppressed proliferation (Figure S1B–E, Supporting Information). To further verify the function of CSN6 in vivo, MiaPaCa‐2 cells expressing shCSN6 were established and then subcutaneously implanted into the flank of nude mice. CSN6 KD markedly inhibited xenograft tumor growth, as evidenced by reduced tumor sizes and weights (Figure 1E). CSN6 KD decreased staining of the cell proliferation marker Ki67 and increased expression of the apoptosis marker cleaved caspase‐3 in these cell‐derived xenografts (CDXs) (Figure 1F). These data suggest that CSN6 plays a pivotal role in promoting pancreatic cancer progression.

In addition, we employed genetically engineered mouse models (GEMMs) carrying alleles that drive spontaneous pancreatic tumorigenesis (including mutations in *Kras* and *Trp53*), conditional *Csn6* KO alleles, and tamoxifen (TAM) inducible pancreas‐specific Cre transgenes (Figure S2A, Supporting Information). Specifically, *Kras^G12D^ Trp53^R172H^* (*LSL‐Kras^G12D/+^; LSL‐Trp53^R172H/+^*) mice^[^ ^23^, ^24^ ^]^ were crossed with *Ptf1a‐Cre^ERT2^* mice^[^ ^25^, ^26^ ^]^ to generate KPP *(LSL‐Kras^G12D/+^; LSL‐Trp53^R172H/+^; Ptf1a‐Cre^ERT2^)* mice, which harbor mutant *Kras* and *Trp53* to induce spontaneous pancreatic tumor formation. The *Csn6^flox/flox^* mice were further introduced into this background by crossing with KPP mice, yielding KPPC (*LSL‐Kras^G12D/+^; LSL‐Trp53^R172H/+^; Ptf1a‐Cre^ERT2^; Csn6^flox/flox^)* mice, thereby enabling pancreas‐specific conditional KO of *Csn6* in a tumor‐prone genetic background (Figure 1G). During the breeding of KPP and KPPC mice, *ZsGreen/tdTomato (G/R) (L‐ZsGreen‐SL‐tdTomato)* reporter mice were crossed with *Ptf1a‐Cre^ERT2^* mice to monitor Cre recombinase activity (Figure S2B, Supporting Information). Following TAM induction via intraperitoneal (i.p.) injection, G/R P *(L‐ZsGreen‐SL‐tdTomato; Ptf1a‐Cre^ERT2^)* mice exhibited red fluorescence in the pancreatic region (Figure S2C, Supporting Information). Ex vivo fluorescence imaging confirmed pancreatic‐specific tdTomato expression resulting from Cre‐mediated recombination, with no detectable signal in other organs (Figure S2C, Supporting Information).

To minimize leaky expression of *Kras^G12D^* and *Trp53^R172H^*, heterozygous KP mice were crossed with either *Ptf1a‐Cre^ERT2^* or *Csn6^flox/flox^* mice. Genotyping PCR was performed on offspring one week after birth (Figure S2D,E, Supporting Information). In KPPC mice (inducible *Csn6* KO), TAM was administered to induce expression of mutant *Kras* and *Trp53* while simultaneously deleting exons 3‐6 of *Csn6*; KPP mice, which carry intact *Csn6*, served as controls (Figure S2A,E, Supporting Information). Both groups were euthanized at 30 weeks of age. Macroscopic examination and hematoxylin & eosin (H&E) staining revealed no tumor formation in KPPC mice (0/3), whereas all KPP mice (3/3) developed solid pancreatic tumors. This represented a directionally significant reduction in tumor incidence (one‐tailed *p* = 0.05; two‐tailed *p* = 0.10) as assessed by Fisher's exact test (Figure 1G,H). Additionally, IHC staining confirmed decreased expression of CSN6 and Ki67 in KPPC mice compared to KPP controls (Figure 1I). These results demonstrate that CSN6 plays a critical role in promoting pancreatic cancer progression.

### 2.2 CSN6 Promotes Ribosome Biogenesis and Protein Synthesis in PDAC

**Figure 2:**
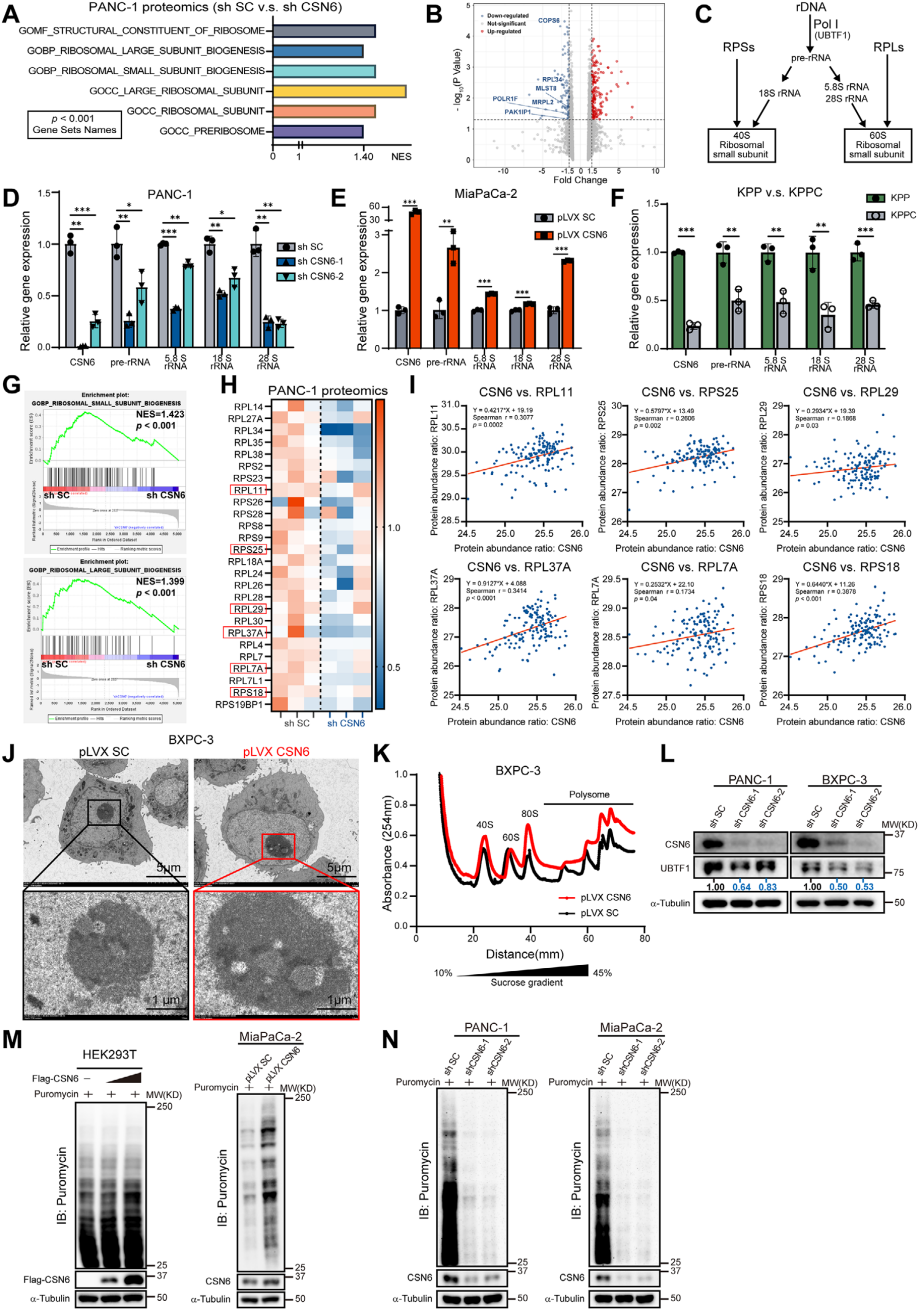
CSN6 regulates ribosome biogenesis and protein synthesis in pancreatic ductal adenocarcinoma (PDAC). A) Gene Ontology (GO) analysis based on quantitative proteomic data reveals enrichment of ribosome biogenesis pathways in control versus CSN6‐KD PANC‐1 cells. B) Differentially expressed proteins between shSC and shCSN6 PANC‐1 cells (|FC| > 1.5, *p* < 0.05). C) Schematic of ribosome biogenesis: rDNA transcription, pre‐rRNA processing, and subunit assembly (RPL/RPS: ribosomal proteins). D–F) qPCR analysis of rRNA levels in CSN6‐KD (D) and CSN6‐OE (E) PDAC cells, and in KPP versus KPPC pancreas tissues (F). G) Proteomic gene set enrichment analysis (GSEA) shows enrichment of ribosome biogenesis pathways in shSC control cells. NES, normalized enrichment score. H) Heatmap of ribosomal proteins (RPs) expression in shSC versus shCSN6 PANC‐1 cells. I) Correlation between CSN6 and various RPs in the CPTAC‐PDA cohort (*n* = 140). J) Representative transmission electron microscopy (TEM) images of nucleolar ultrastructure in control and CSN6‐OE BXPC‐3 cells. K) Ribosome profiling of BXPC‐3 cells overexpressing CSN6. pLVX SC or pLVX CSN6 cells were lysed and subjected to sucrose gradient centrifugation; sucrose gradient profiles were monitored by measuring absorbance at 254 nm (A_254)_. Positions of 40S, 60S, 80S, and polysomes in the sucrose gradient are labeled. L) Reduced UBTF1 protein expression in CSN6‐KD PDAC cells. Protein expression levels were assessed by immunoblotting and quantified using ImageJ. M,N) Puromycin incorporation assays showing altered translation activity in CSN6‐OE (M) and CSN6‐KD (N) cells. Cells were incubated with puromycin (10 µg mL^−1^) for 30 min, lysed, and subjected to immunoblotting with an anti‐puromycin antibody. All quantitative data are presented as mean ± SD. Statistical analysis was performed using one‐way ANOVA (D–F) and Spearman's rank correlation (I). Significance is indicated as follows: ^*^ *p* < 0.05, ^**^ *p* < 0.01, ^***^ *p* < 0.001.

To elucidate the molecular mechanism by which CSN6 promotes pancreatic cancer progression, we performed quantitative proteomic analysis (LC‐MS) using PANC‐1 cells treated with either shSC (Scramble) or shCSN6 (Figure S3A, Supporting Information). Principal component analysis (PCA) indicated that CSN6 KD markedly altered the global protein expression profile (Figure S3B, Supporting Information). We then conducted gene set enrichment analysis (GSEA) on the proteomic data after mapping protein‐to‐gene identifiers to identify biological processes influenced by CSN6. GSEA revealed that multiple ribosome biogenesis‐associated pathways were downregulated in the shCSN6 group, including pre‐ribosome and ribosome large/small subunit biogenesis (**Figures** **2**A and S3C, Supporting Information). The volcano plot highlighted the most significantly altered gene products after CSN6 KD. Notably downregulated proteins included POLR1F, which encodes a subunit of DNA‐dependent RNA polymerase I responsible for synthesizing ribosomal RNA precursors (pre‐rRNAs), and RPL34, which encodes a component of the 60S large ribosomal subunit (Figure 2B). Ribosome biogenesis encompasses the synthesis of ribosomal RNAs (rRNAs), their processing and modification, the transport of ribosomal proteins (RPs), and assembly and nuclear export of the 40S/60S subunits (Figure 2C), all processes essential for cellular protein synthesis. Structurally, the 40S small subunit contains the 18S rRNA and 33 small ribosomal proteins (RPSs), while the 60S large subunit comprises 5S, 5.8S, and 28S rRNAs along with 47 large ribosomal subunit proteins (RPLs).^[^ ^27^ ^]^

Given the central role of rRNAs and RPs in ribosome biogenesis, we evaluated the effect of CSN6 perturbation on rRNA levels in PDAC cells using quantitative PCR (qPCR). CSN6 KD reduced the levels of pre‐rRNA, 5.8S rRNA, 18S rRNA, and 28S rRNA, whereas CSN6 OE increased them (Figure 2D,E). Consistent with these in vitro findings, pancreatic tissues from KPPC mice (with *Csn6* KO) exhibited lower rRNA expression compared to those from KPP controls (Figure 2F), corroborating the role of CSN6 in ribosome biogenesis in vivo. GSEA and a heat map of proteomic data showed that CSN6 KD suppressed the biogenesis of ribosomal small and large subunits by affecting multiple RPLs and RPSs (Figure 2G,H). For example, protein levels of RPs such as RPL11, RPL25, RPL37A, RPL29, RPL7A, and RPS18 were reduced in the CSN6 KD group. Further protein co‐expression analysis of the CPTAC_PDA dataset confirmed a positive correlation between CSN6 expression and these ribosomal components (Figure 2I).

Transmission electron microscopy (TEM) revealed an enlarged nucleolus in CSN6‐OE PDAC cells compared with controls, suggesting enhanced ribosome biogenesis (Figure 2J). Ribosome profiling further demonstrated that ectopic expression of CSN6 increased the levels of 40S, 60S, and 80S subunits as well as polysomes, suggesting enhanced global protein translation (Figure 2K). Since UBTF1 (upstream binding transcription factor 1) is a master regulator of ribosomal RNA transcription,^[^ ^28^, ^29^ ^]^ we investigated whether CSN6 influences rRNA expression through UBTF1. Indeed, CSN6 KD led to decreased expression of UBTF1 protein in pancreatic cancer cell lines (Figure 2L), suggesting a functional link between CSN6 and UBTF1.

To further verify the downstream biological consequence of CSN6‐mediated ribosome biogenesis, we evaluated protein synthesis using a puromycin incorporation assay. Ectopic CSN6 expression enhanced protein synthesis (Figure 2M), while CSN6 KD markedly suppressed it (Figure 2N). Together, these data demonstrate that CSN6 facilitates ribosome biogenesis and promotes protein synthesis by enhancing rRNA transcription via UBTF1 and by sustaining the expression of RPs.

### 2.3 CSN6 Binds to NPM1 and Stabilizes it by Modulating Ubiquitination, Thereby Promoting NPM1‐Mediated Ribosome Biogenesis and Cell Growth

**Figure 3:**
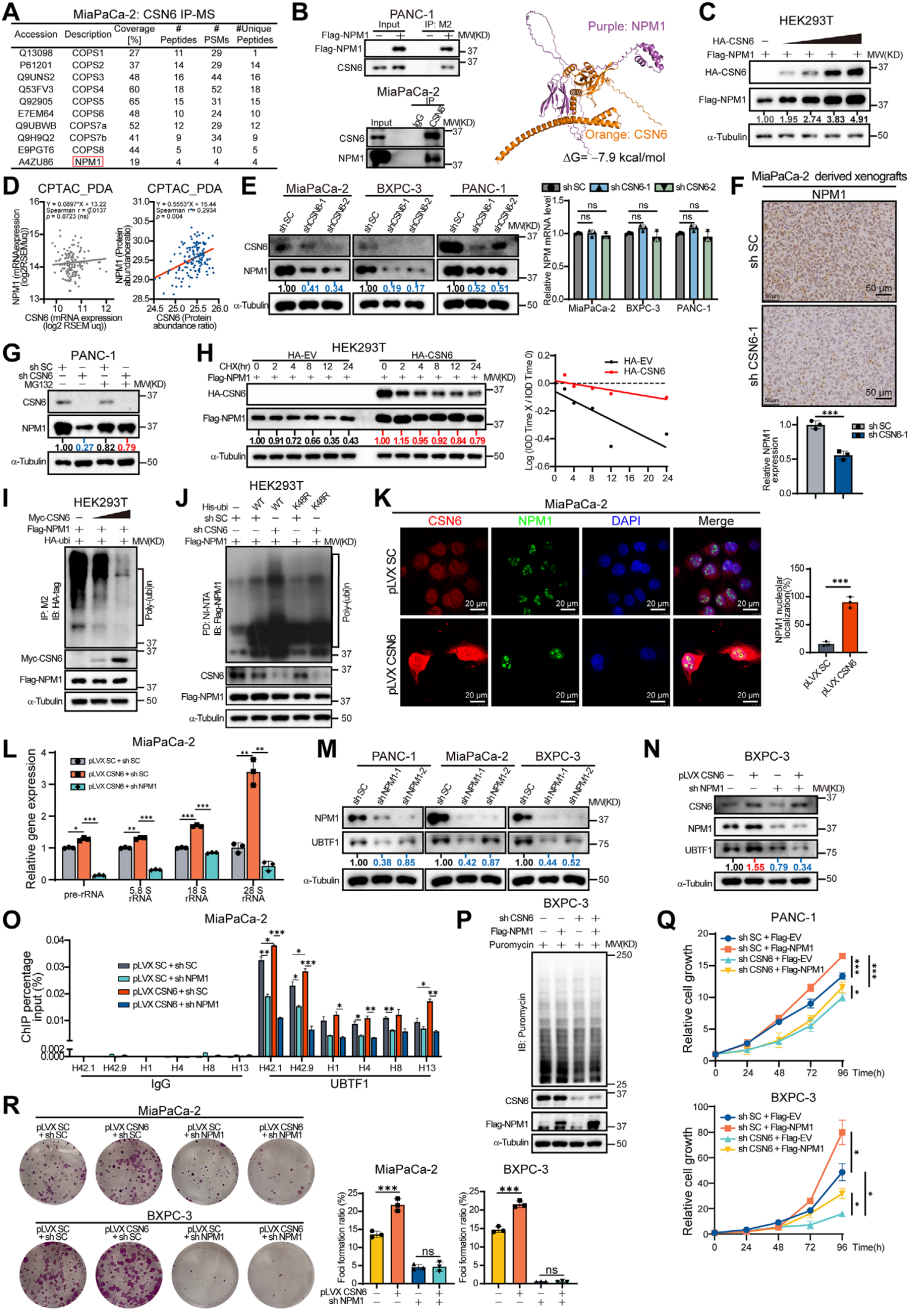
CSN6 stabilizes NPM1 via ubiquitination to drive ribosome biogenesis and tumor growth. A) CSN6‐interacting proteins were identified by mass spectrometry (MS) in MiaPaCa‐2 cells. B) Exogenous and endogenous CSN6‐NPM1 interactions in pancreatic ductal adenocarcinoma (PDAC) cells (co‐IP assays, left). Co‐IP was performed using anti‐Flag M2 beads for exogenous interactions or specific antibodies plus Protein A/G agarose beads for endogenous interactions. The Flag‐EV (empty vector) or IgG served as the negative control. Schematic of CSN6‐NPM1 binding (right). Computational modeling of CSN6‐NPM1 interactions was performed by AlphaFold3, followed by visualization with PyMOL v2.5. C) CSN6‐OE upregulated NPM1 protein expression in HEK293T cells. Cells were transfected with the indicated constructs and immunoblotted with the indicated antibodies. D) Positive correlation of CSN6 and NPM1 proteins in CPTAC‐PDA cohort (*n* = 140, right). No correlation was observed at the mRNA level (left). E) CSN6 depletion decreased NPM1 protein stability (left) without affecting its mRNA (right) in three independent PDAC cell lines. F) Representative immunohistochemical (IHC) staining (top) and ImageJ quantification (bottom) of NPM1 in xenograft tumors derived from Figure 1E. G) Proteasome inhibitor MG132 (10 × 10^−6^ m, 6 h) rescued NPM1 downregulation caused by CSN6 KD. H) CSN6‐OE extended NPM1 half‐life. Cells were treated with cycloheximide (CHX, 20 µg mL^−1^) for the indicated time points following CSN6 KD, followed by immunoblotting to illustrate protein degradation kinetics. I) Dose‐dependent decrease in NPM1 ubiquitination following CSN6‐OE. HEK293T cells were transfected with the indicated plasmids and treated with MG132 (10 × 10^−6^ m, 6 h) prior to anti‐Flag M2 pull‐down and immunoblotting with the indicated antibodies. J) CSN6 mediated K48‐linked NPM1 polyubiquitination. Cells transfected with the indicated constructs were treated with MG132 (10 × 10^−6^ m) for 6 h before harvest. The cell lysates were pulled down with Ni‐NTA beads and immunoblotted with the indicated antibodies. K) IF analysis of NPM1 in CSN6‐OE PDAC cells (left). Quantification of NPM1 nucleolar localization frequency (right). L) qPCR revealing rRNA expression profiles under conditions of CSN6‐OE versus CSN6‐OE plus NPM1‐KD. M) NPM1 depletion reduced UBTF1 protein levels across PDAC cell lines. N) Immunoblot analysis of UBTF1 expression in BXPC‐3 cells with pLVX‐SC/CSN6 and SC/NPM1 shRNAs. O) ChIP‐qPCR evaluating UBTF1 occupancy on rDNA transcriptional regulatory regions under conditions of CSN6 OE and/or NPM1 knockdown (KD). P) Puromycin incorporation assay revealed global translation rates in CSN6‐KD ± Flag‐NPM1‐OE BXPC‐3 cells. Cells incubated with puromycin (10 µg mL^−1^) for 30 min were lysed and subjected to immunoblotting analysis with an anti‐puromycin antibody. Q) Proliferation kinetics of PDAC cells with CSN6‐KD plus NPM1‐OE were measured by CCK‐8 assay. R) Foci formation assay evaluating PDAC proliferation under CSN6‐OE plus NPM1‐KD. Data are presented as the mean ± SD. Statistical analysis was performed using Spearman's rank correlation (D), one‐way ANOVA (E,L,O,R), Student's *t*‐test (F,K), or two‐way ANOVA (Q). Statistical significance in the figures is indicated as follows: ns for *p* > 0.05, ^*^ *p* < 0.05, ^**^ *p* < 0.01, ^***^ *p* < 0.001.

To investigate the mechanism by which CSN6 promotes ribosome biogenesis, we screened for its interacting protein partners using immunoprecipitation coupled with mass spectrometry (IP‐MS) (Figure S4A, Supporting Information). This approach identified multiple proteins co‐immunoprecipitated with CSN6, including subunits of the COP9 signalosome complex (Table S1, Supporting Information). Among these interactors, we focused on nucleophosmin (NPM1), a multifunctional nucleolar phosphoprotein known to promote cell survival, as a candidate (**Figure** **3**A). NPM1 is an abundant oligomeric protein in the granular component of the nucleolus and plays multiple important roles in ribosome biogenesis, including rRNA synthesis and processing, ribosomal protein transport, and ribosomal subunit assembly and export.^[^ ^30^ ^]^

Data mining of the TCGA_PDAC dataset revealed that NPM1 OE in pancreatic cancer was associated with unfavorable clinical outcomes (Figure S4B,C, Supporting Information). Functional assays demonstrated that NPM1 promotes pancreatic cancer cell proliferation, as NPM1 KD significantly inhibited cell growth and foci formation (Figure S4D,E, Supporting Information). GSEA based on the PDAC dataset GSE28735 further supported the functional involvement of NPM1 in ribosome biogenesis (Figure S4F, Supporting Information). Consistent with this, NPM1 KD in two PDAC cell lines led to reduced rRNA levels (Figure S4G, Supporting Information). These data demonstrate the oncogenic role of NPM1 in PDAC and its promotive role in ribosome biogenesis.

Co‐immunoprecipitation (co‐IP) experiments confirmed the physical interaction between CSN6 and NPM1 in PDAC cell lines, a finding further corroborated by molecular docking simulations that predicted a structural model of their binding (Figure 3B and Figure S4H, Supporting Information). To explore the functional relevance of this interaction, we evaluated NPM1 expression under CSN6 modulation. Ectopic expression of CSN6 resulted in elevated NPM1 protein levels (Figure 3C). Analysis of the CPTAC_PDA dataset showed no correlation between CSN6 and NPM1 mRNA expression, whereas a significant positive correlation was observed at the protein level, suggesting post‐transcriptional regulation (Figure 3D). KD of CSN6 further validated this effect, reducing NPM1 protein levels without affecting its mRNA expression in three PDAC cell lines (Figure 3E) and nude mouse xenografts (Figure 3F). These data indicate that CSN6 regulates NPM1 protein expression post‐transcriptionally.

The decrease in NPM1 protein expression mediated by CSN6 KD was rescued by the proteasome inhibitor MG132, suggesting that the ubiquitin‐proteasome system is involved in this regulatory process (Figure 3G). Moreover, cycloheximide (CHX) chase assays demonstrated that CSN6 decreases the turnover rate of NPM1 (Figure 3H). CSN6 OE reduced the ubiquitination level of NPM1, as shown in co‐transfection and pull‐down experiments (Figure 3I). Interestingly, although CSN6 KD increased NPM1 ubiquitination, this effect was attenuated upon expression of a His‐tagged K48R ubiquitin mutant, suggesting the involvement of K48‐linked polyubiquitination (Figure 3J). Hence, CSN6 stabilizes NPM1 by inhibiting its ubiquitination.

Immunofluorescence (IF) analysis in MiaPaCa‐2 cells revealed that ectopic expression of CSN6 promoted the nucleolar localization of NPM1 (Figure 3K). We next investigated whether NPM1 is required for CSN6‐mediated rRNA upregulation. Indeed, NPM1 KD attenuated the increase in rRNAs induced by CSN6 OE (Figure 3L). Since we had previously found that NPM1 was crucial for UBTF1 expression and basal rRNA regulation (Figure 3M and Figure S4G, Supporting Information), we hypothesized that CSN6 might exert its effect by modulating UBTF1 through NPM1. Consistent with this, CSN6‐mediated UBTF1 upregulation was compromised upon NPM1 KD (Figure 3N). Further chromatin immunoprecipitation quantitative PCR (ChIP‐qPCR) analysis showed that CSN6 OE increased the occupancy of UBTF1 at the rDNA promoter region, an effect that NPM1 KD reversed (Figure 3O and Figure S4I, Supporting Information). Together, these results delineate a linear pathway in which CSN6 upregulates UBTF1 through NPM1 to promote rRNA transcription.

Subsequent puromycin incorporation assays indicated that CSN6 KD inhibited protein synthesis, and this inhibition could be rescued by NPM1 OE (Figure 3P). Collectively, these data demonstrate that CSN6‐NPM1 interaction plays a pivotal role in rRNA transcription and ribosome biogenesis. Accordingly, the growth inhibition mediated by CSN6 KD was reversed by NPM1 OE, as shown by CCK‐8 analysis (Figure 3Q). Foci formation assays indicated that the proliferative effect induced by CSN6 was compromised following NPM1 depletion (Figure 3R). Taken together, these findings demonstrate that the CSN6‐NPM1 axis promotes ribosome biogenesis and enhances cell proliferation.

### 2.4 DCAF1 is a CSN6‐Regulated E3 Ubiquitin Ligase and is Responsible for NPM1 Ubiquitination and its Consequent Destabilization

**Figure 4:**
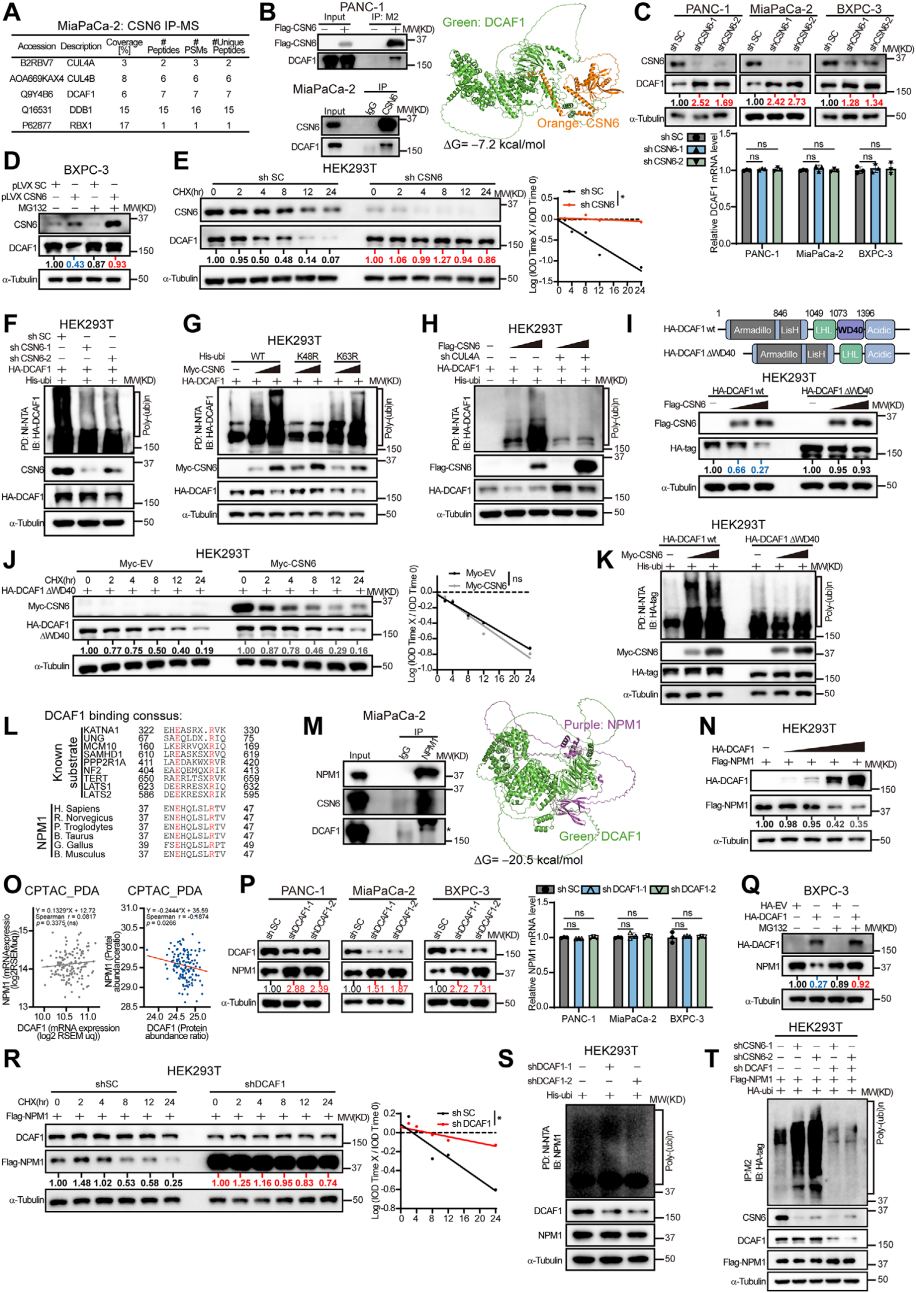
CSN6 stabilizes NPM1 through antagonizing DCAF1‐mediated NPM1 ubiquitination. A) Mass spectrometry (MS) analysis of the CSN6 interactome identified CRL4 complex subunits. B) Exogenous and endogenous CSN6‐DCAF1 interaction in pancreatic ductal adenocarcinoma (PDAC) cells (co‐IP assays, left). Co‐IP was performed using anti‐Flag M2 beads (exogenous) or antibodies plus Protein A/G agarose beads (endogenous). The Flag‐EV (empty vector) or IgG served as the negative control. Schematic of CSN6‐DCAF1 binding (right). Computational modeling of CSN6‐DCAF1 interactions by AlphaFold3 followed by PyMOL v2.5 visualization. C) CSN6‐KD increased DCAF1 protein levels (upper) without affecting its mRNA levels (lower) in three independent PDAC cell lines. D) Proteasome inhibitor MG132 (10 µm, 6 h) reversed CSN6‐mediated DCAF1 degradation. E) CSN6 depletion prolonged DCAF1 half‐life in CHX chase experiment. Cells were treated with CHX (20 µg mL^−1^) for the indicated times, followed by immunoblotting illustration of protein degradation kinetics. F) CSN6‐KD reduced DCAF1 polyubiquitination. HEK293T cells were transfected as indicated and treated with MG132, followed by Ni‐NTA pull‐down and immunoblotting. G) His‐tagged single‐lysine mutants of ubiquitin reveal that CSN6‐OE enhances K48‐linked polyubiquitination of DCAF1. HEK293T cells were transfected as indicated and treated with MG132 (10 µm, 6 h), followed by Ni‐NTA pull‐down and immunoblotting. H) Cul4A‐KD ubiquitin experiment reveals CRL4 dependency for CSN6‐mediated DCAF1 ubiquitination. I) CSN6 destabilized WT DCAF1 but not WD40 mutant (ΔWD40). DCAF1 domain structure (upper) and immunoblot experiment (lower). J) CHX chase experiment confirming ΔWD40 mutant resistance to CSN6‐mediated degradation. Cells were treated with CHX (20 µg mL^−1^) for the indicated times, followed by immunoblotting illustrating protein degradation kinetics. K) Comparative ubiquitination assay identifying WD40 domain requirement in CSN6‐induced DCAF1 polyubiquitination. HEK293T cells were transfected as indicated, treated with MG132, followed by Ni‐NTA pull‐down and immunoblotting. L) Evolutionary conservation of DCAF1‐binding motif in NPM1. Conserved aa residues in KATNA1/NF2/LATS1/2 recognition motif. M) Endogenous co‐IP of NPM1‐DCAF1‐CSN6 interaction (left). Co‐IP was performed using antibodies plus Protein A/G agarose beads. IgG served as a negative control. Interaction modeling of the NPM1‐DCAF1 complex (right). N) Immunoblotting revealed DCAF1‐OE decreased NPM1 expression in a dose‐dependent manner. O) Negative protein correlation between DCAF1 and NPM1 in CPTAC‐PDA cohort (*n* = 140, right), but no correlation was observed at the mRNA level (left). P) DCAF1 depletion increased NPM1 protein stability (left) without affecting its mRNA (right) in three independent PDAC cell lines. Q) Proteasome inhibitor MG132 blocked DCAF1‐mediated NPM1 degradation. R) CHX chase experiment demonstrating DCAF1‐KD extended NPM1 half‐life. Cells were treated with CHX (20 µg mL^−1^) for the indicated time points in control or DCAF1‐KD cells, followed by immunoblotting analysis. S) DCAF1‐KD reduced NPM1 polyubiquitination. HEK293T cells were transfected as indicated and treated with MG132 for 6 h prior to harvesting. Cell lysates were subjected to pull‐down with Ni‐NTA beads and immunoblotted with the indicated antibodies. T) DCAF1's role in CSN6‐mediated NPM1 ubiquitination. HEK293T cells were transfected as indicated and treated with MG132 for 6 h before harvesting. Cell lysates were then pulled down by anti‐Flag M2 beads and immunoblotted with the indicated antibodies. Data are presented as the mean ± SD. Statistical analysis was performed using one‐way ANOVA (C,P), two‐way ANOVA (E,J,R), or Spearman's rank correlation analysis (O). Statistical significance is indicated as follows: ns for *p* > 0.05, *p < 0.05.

To further investigate the mechanism of CSN6‐mediated reduction in NPM1 ubiquitination, we analyzed CSN6‐associated proteins in our IP‐MS dataset and identified several ubiquitin regulatory components, including Cullin4A/B, DDB1, RBX1, and DCAF1 (**Figure** **4**A). Given that DCAF1 serves as the substrate receptor of the Cullin4 complex and considering CSN6's established role in regulating Cullin substrate receptors, we investigated whether CSN6 facilitates NPM1 ubiquitination through DCAF1. Co‐IP experiments confirmed that CSN6 interacts with DCAF1, and molecular docking predicted a structural model for their interaction (Figure 4B and Figure S5A, Supporting Information). CSN6 KD increased the steady‐state protein level of DCAF1 in three PDAC cell lines as well as in our xenograft models (Figure 4C and Figure S5B, Supporting Information), without altering its mRNA expression, suggesting regulation at the post‐transcriptional level. Furthermore, the decrease in DCAF1 protein expression mediated by CSN6 OE was rescued by the proteasome inhibitor MG132, implicating the ubiquitin‐proteasome pathway in this process (Figure 4D). CHX chase assays indicated that CSN6 KD prolonged the half‐life of DCAF1 (Figure 4E). Further His‐tagged ubiquitination assays showed that CSN6 KD decreased DCAF1 ubiquitination (Figure 4F), while CSN6 OE enhanced K48‐linked polyubiquitination of DCAF1 (Figure 4G and Figure S5C, Supporting Information). These data demonstrate that CSN6 negatively regulates DCAF1 by enhancing its ubiquitination.

Since DCAF1 is a component of Cullin4‐Ring Ligases (CRL4) complexes, we hypothesized that CSN6 might promote DCAF1 ubiquitination through Cullin4. Indeed, Cullin4 KD attenuated CSN6 OE‐mediated DCAF1 ubiquitination (Figure 4H), suggesting that CSN6‐induced ubiquitination of DCAF1 is Cullin4‐dependent. As the WD40 domain is indispensable for DCAF1's incorporation into the CRL4 complex and its subsequent function in substrate ubiquitination, we generated a WD40 deletion mutant, DCAF1ΔWD40. CSN6 OE dramatically downregulated wild‐type (wt) DCAF1 protein but had no effect on the DCAF1ΔWD40 mutant (Figure 4I). Consistently, CSN6 did not alter the protein turnover rate of the DCAF1ΔWD40 mutant (Figure 4J). Furthermore, although CSN6 enhanced ubiquitination of wild‐type DCAF1, it failed to promote ubiquitination of the DCAF1ΔWD40 mutant (Figure 4K). Taken together, these results demonstrate that CSN6‐mediated ubiquitination and degradation of DCAF1 depend on Cullin4 and require an intact WD40 domain, which is essential for DCAF1 auto‐ubiquitination.

Protein sequence alignment revealed that NPM1 contains a conserved motif matching the binding consensus of known DCAF1 substrates (Figure 4L). Endogenous co‐IP assays in MiaPaCa‐2 cells confirmed that NPM1 interacts with both CSN6 and DCAF1 (Figure 4M). Molecular docking further predicted a structural model of the interaction between the E3 ligase DCAF1 and its putative substrate NPM1 (Figure 4M and Figure S5D, Supporting Information). Domain mapping indicated that DCAF1 binds NPM1 at its N‐terminal and aromatic regions (Figure S5E, Supporting Information). DCAF1 OE decreased the steady‐state expression of NPM1 (Figure 4N). Analysis of the CPTAC_PDA dataset showed a negative correlation between DCAF1 and NPM1 protein abundance, with no correlation observed at the mRNA level (Figure 4O). Consistent with this, DCAF1 KD in three PDAC cell lines increased NPM1 protein expression without affecting its mRNA, further supporting post‐transcriptional regulation (Figure 4P and Figure S5F, Supporting Information). DCAF1‐induced NPM1 downregulation was rescued by MG132, confirming proteasome‐dependent degradation (Figure 4Q). CHX chase assays indicated that DCAF1 KD decelerated the turnover of NPM1 (Figure 4R). Additionally, DCAF1 KD led to a decrease in the ubiquitination of NPM1 (Figure 4S), suggesting that DCAF1 acts as an E3 ubiquitin ligase for NPM1.

To validate the involvement of DCAF1 as an E3 ligase in CSN6‐mediated regulation of NPM1 ubiquitination, we performed CSN6 KD in combination with DCAF1‐targeting shRNA. CSN6 KD‐mediated NPM1 ubiquitination increase was attenuated when DCAF1 was concurrently depleted (Figure 4T), confirming a functional CSN6‐DCAF1‐NPM1 regulatory axis. Collectively, these results indicate that CSN6 promotes the auto‐ubiquitination and degradation of DCAF1. As DCAF1 is an E3 ligase for NPM1, its degradation attenuates DCAF1‐mediated NPM1 ubiquitination and ultimately stabilizes NPM1 protein.

### 2.5 Gemcitabine‐Resistant PDAC Cells have High Expression of CSN6, Concurrent with Increased Ribosome Biogenesis and Protein Synthesis

**Figure 5:**
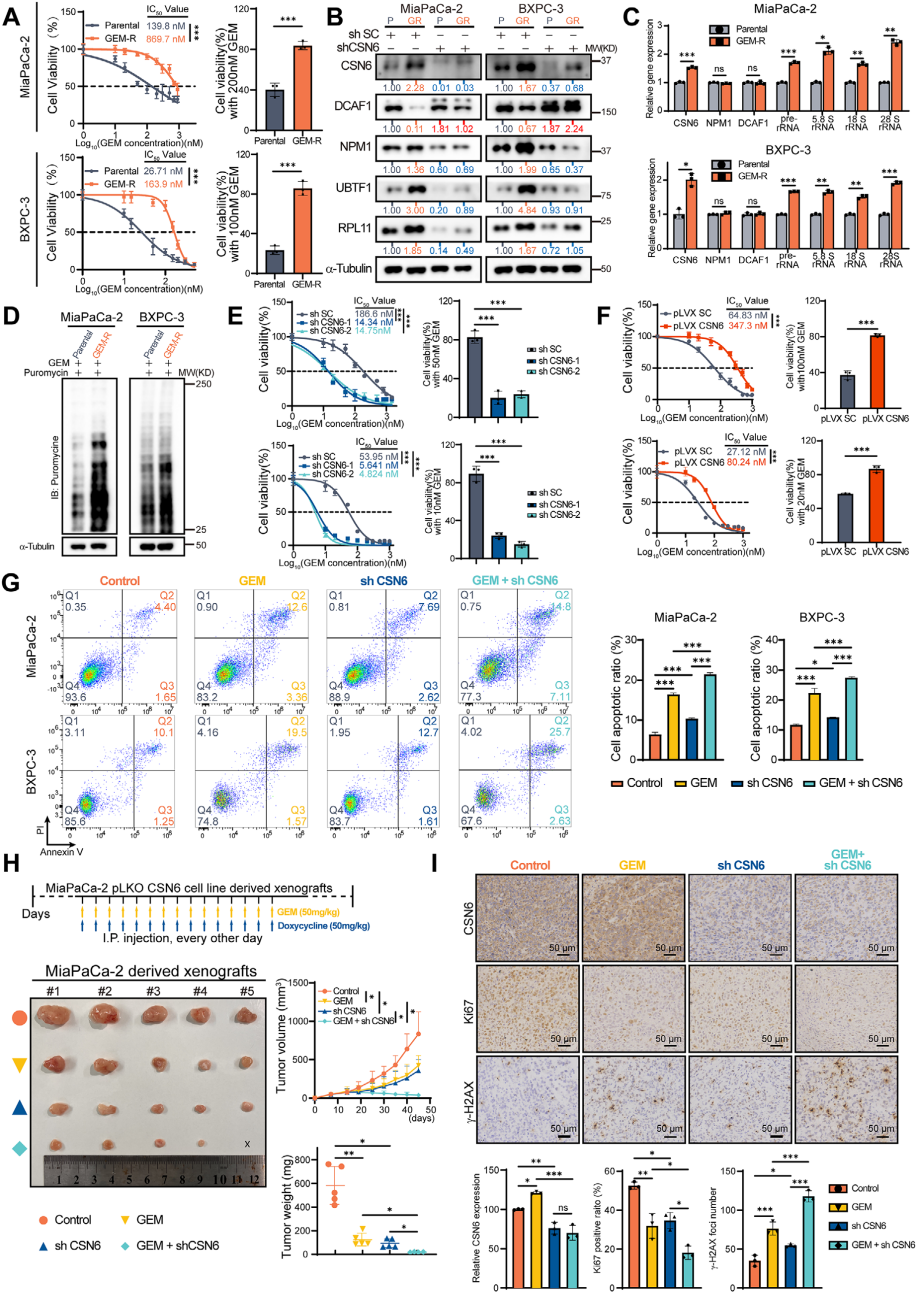
CSN6 overexpression (OE) confers gemcitabine resistance in pancreatic ductal adenocarcinoma (PDAC). A) Dose‐response curves of gemcitabine‐resistant (GEM‐R) PDAC cells compared to parental cells. IC_50_ was compared by nonlinear regression with labeled values. B) Immunoblots showing CSN6/NPM1 upregulation and DCAF1 downregulation in GEM‐R cells (left two lanes), which were reversed by CSN6‐KD (right two lanes). C) qPCR detecting CSN6/NPM1/DCAF1/rRNA levels in GEM‐R versus parental cells. D) Puromycin incorporation assay demonstrating enhanced nascent protein synthesis in GEM‐R cells. Cells were incubated with puromycin (10 µg mL^−1^) for 30 min before being lysed for immunoblotting analysis. E) Increased gemcitabine sensitivity in CSN6‐KD PDAC cells compared with control cells. IC_50_ was compared by nonlinear regression with labeled values. F) Gemcitabine resistance in CSN6‐OE PDAC cells compared to control cells. IC_50_ was compared by nonlinear regression with labeled values. G) CSN6‐KD and gemcitabine treatment induced apoptosis in PDAC cells. Annexin V/PI flow cytometry was performed (left), with quantified apoptotic rates shown (right). The cell apoptotic ratio represents the combined percentage of Q2 (late apoptotic cells) and Q3 (early apoptotic cells). H) Experimental design for inducible CSN6‐KD in MiaPaCa‐2 cells and xenograft model (top). Representative image of endpoint xenografts (lower left), and tumor growth curve and final tumor weights (lower right) are shown. I) Immunohistochemical (IHC) staining of CSN6, Ki67, and γ‐H2AX in xenograft tumors (top), and quantitative analysis of staining intensities using ImageJ (bottom). Data are presented as the mean ± SD. Statistical analysis was performed using nonlinear regression for IC_50_ comparison (A, E, F‐left), Student's t‐test (A, F‐right), one‐way ANOVA (C, E, G, H‐tumor weight, I), or two‐way ANOVA (J‐tumor volume). Statistical significance is indicated as follows: ns for *p* > 0.05, ^*^ *p* < 0.05, ^**^ *p* < 0.01, ^***^ *p* < 0.001.

Gemcitabine remains a cornerstone in the chemotherapy of pancreatic cancer. However, its clinical efficacy is often limited by suboptimal response rates. Based on previous reports implicating NPM1 in drug resistance,^[^ ^31^ ^]^ we hypothesized that CSN6 and its downstream effector NPM1 might modulate gemcitabine response in PDAC. To investigate this, we established two gemcitabine‐resistant (GEM‐R) PDAC cell lines through prolonged exposure to a non‐lethal dose of gemcitabine over 6 months (**Figure** **5**A). Western blot analysis revealed upregulation of CSN6 in the GEM‐R cells, along with elevated expression of NPM1, UBTF1, and RPL11, and reduced expression of DCAF1 (Figure 5B). These results suggest that proteins positively regulated by CSN6 are elevated in gemcitabine‐resistant cells. To further investigate the link between ribosome biogenesis and gemcitabine resistance, we measured rRNA expression using qPCR. Both GEM‐R cell lines exhibited consistent upregulation of pre‐rRNA, 5.8S rRNA, 18S rRNA, and 28S rRNA compared to parental (P) cells (Figure 5C). Additionally, GEM‐R cells display enhanced global protein synthesis activity (Figure 5D). Collectively, these results indicate that the CSN6‐NPM1 axis and downstream ribosome biogenesis are augmented in gemcitabine‐resistant PDAC cells, potentially contributing to gemcitabine resistance.

To further investigate the role of CSN6 in gemcitabine resistance, PDAC cells were infected with lentivirus expressing either shSC or shCSN6 and treated with a concentration gradient of gemcitabine. Cell viability was measured and used to plot the IC_50_ curve. CSN6 KD resulted in a pronounced leftward shift of the IC_50_ curves in two PDAC cell lines, indicating enhanced gemcitabine sensitivity (Figure 5E). The bar graph further illustrated significantly reduced cell viability in the CSN6 KD group at specific gemcitabine concentrations (Figure 5E). This reduction correlated with the downregulation of NPM1, UBTF1, and RPL11 (Figure 5B). Conversely, CSN6 OE conferred gemcitabine resistance, as evidenced by the rightward shift in the IC_50_ curve and reduced cell death after gemcitabine treatment (Figure 5F). Flow cytometry confirmed that CSN6 KD promoted apoptosis and increased sensitivity to gemcitabine compared to the control group (Figure 5G). These data suggest that CSN6 mediates gemcitabine resistance and that KD of CSN6 sensitizes PDAC cells to gemcitabine.

CDA and RRM1/2 are key factors associated with gemcitabine resistance.^[^ ^32^ ^]^ CDA promotes gemcitabine metabolic inactivation, while RRM1 and RRM2 regulate pyrimidine metabolism and deoxyribonucleotides supply, facilitating DNA repair and conferring gemcitabine resistance.^[^ ^32^ ^]^ We hypothesized that CSN6‐regulated ribosome biogenesis might contribute to gemcitabine resistance by enhancing the translation of CDA, RRM1, and RRM2. To test this, monosome and polysome fractions from CSN6‐OE and control cells were isolated and subjected to RT‑qPCR analysis. The proportion of mRNA in polysomes reflects its translational activity. Compared to controls, CDA/RRM1/RRM2 mRNAs showed increased polysome occupancy in CSN6‐OE cells, indicating enhanced translation efficiency (Figure S6A¸ Supporting Information). Notably, KD of CDA attenuated CSN6‐induced gemcitabine resistance, suggesting that CDA contributes to the CSN6‐mediated resistant phenotype (Figure S6B, Supporting Information). These data indicate that CSN6‐enhanced ribosome biogenesis promotes gemcitabine resistance, likely mediated by the enhanced translation of proteins associated with drug resistance.

We next established a subcutaneous xenograft model of pancreatic cancer in nude mice by injecting doxycycline (DOX)‐inducible CSN6‐KD cells to evaluate whether CSN6 depletion enhanced gemcitabine efficacy in vivo. Compared with the control group, either CSN6 KD or gemcitabine treatment alone reduced tumor growth, size, and weight. However, a more pronounced inhibitory effect was observed in the group that received combined CSN6 KD and gemcitabine treatment, in the absence of systemic toxicity (Figure 5H and Figure S6B, Supporting Information). IHC staining demonstrated that the combination treatment enhanced tumor growth inhibition and DNA damage, as indicated by decreased Ki67 staining and increased γ‐H2AX signal (Figure 5I). Together, these data indicate that CSN6 OE strengthens ribosome biogenesis and translation of gemcitabine resistance‐associated proteins (e.g., CDA, RRM1, RRM2), which partially explains ribosome biogenesis‐mediated chemoresistance. Furthermore, CSN6 KD facilitated gemcitabine‐induced toxicity both in vitro and in vivo.

### 2.6 The Combination Treatment of the NPM1 Inhibitor NSC348884 and Gemcitabine Mitigated Tumor Growth in a CSN6‐High Pancreatic Cancer Model

**Figure 6:**
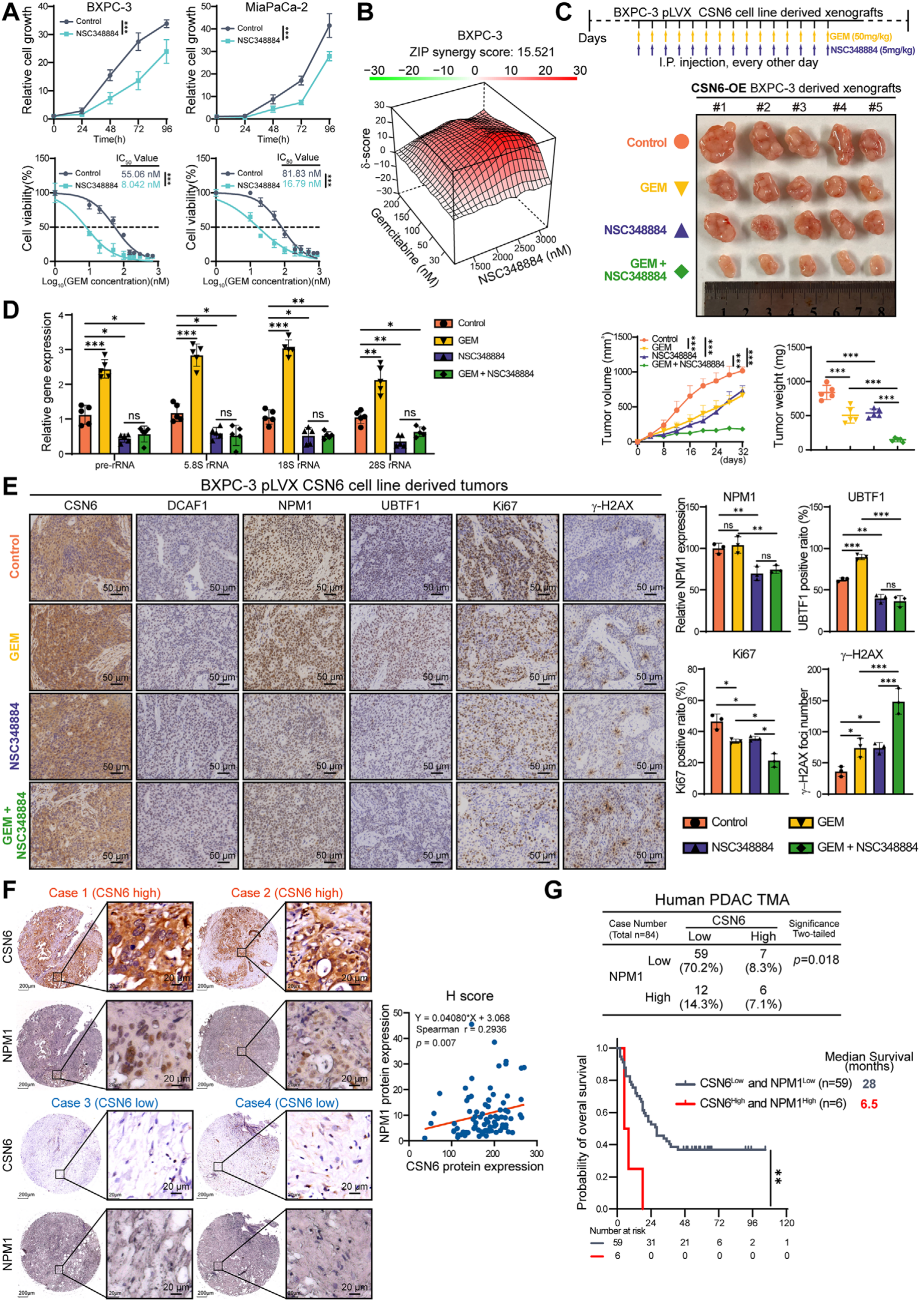
Pharmacological inhibition of NPM1 overcomes CSN6‐mediated gemcitabine resistance. A) Anti‐proliferative effects of NSC348884 in pancreatic ductal adenocarcinoma (PDAC) cells by CCK‐8 assay (top). NSC348884 chemosensitized PDAC cells to gemcitabine treatment with IC_50_ values compared by nonlinear regression (bottom). B) NSC348884 synergized with gemcitabine in PDAC cells. The synergistic effect was assessed using SynergyFinder. C) Xenograft model using CSN6‐overexpressing BXPC‐3 cells (upper panel). Representative images of tumor specimens (middle), longitudinal measurements of tumor volume (lower left), and final tumor weights from the indicated treatment groups (lower right) are shown. D) qPCR detecting rRNA levels in xenograft tumors treated with GEM plus NSC348884. E) Immunohistochemical (IHC) staining of CSN6, DCAF1, NPM1, UBTF1, Ki67, and γ‐H2AX (left), and quantification of NPM1, UBTF1, Ki67, and γ‐H2AX staining intensities using ImageJ (right). F) IHC staining of CSN6 and NPM1 on a PDAC tissue microarray. Representative images from cases with high (Cases 1‐2) or low (Cases 3‐4) CSN6 expression (left). Scatter plot illustrating the positive correlation between CSN6 and NPM1 H‐scores (*n* = 84; right). G) Overall survival Kaplan‐Meier curves for patients from the TMA cohort, grouped based on combined high (CSN6^high^/NPM1^high^) or low (CSN6^low^/NPM1^low^) expression of CSN6 and NPM1. The cutoff for high/low expression was determined using receiver operating characteristic analysis. Data are presented as the mean ± SD. Statistical analysis was performed using two‐way ANOVA (A‐top, C‐tumor volume), nonlinear regression for IC_50_ comparison (A‐bottom), one‐way ANOVA (C‐tumor weight, D, E), Spearman's rank correlation analysis (F‐right), chi‐square test (G‐top), or log‐rank test in (G‐bottom). Statistical significance is indicated as follows: ns for *p* > 0.05, ^*^ *p* < 0.05, ^**^ *p* < 0.01, ^***^ *p* < 0.001.

NSC348884, a small‐molecule inhibitor targeting NPM1, disrupts its biological function via oligomerization interference (Figure S7A, Supporting Information).^[^ ^33^, ^34^ ^]^ To assess the therapeutic potential of targeting the CSN6‐NPM1 axis for suppressing PDAC progression and overcoming gemcitabine resistance, we therefore treated PDAC cell lines with NSC348884. This inhibitor significantly suppressed proliferation in two PDAC cell lines (**Figure** **6**A). A leftward shift in the IC_50_ curve indicated that NSC348884 sensitized PDAC cells to gemcitabine (Figure 6A). Furthermore, synergy analysis using SynergyFinder confirmed that NSC348884 acts synergistically with gemcitabine in PDAC cells (Figure 6B).

To determine the clinical significance of targeting the CSN6‐NPM1 axis with NSC348884, we subcutaneously implanted CSN6‐OE BXPC‐3 cells into nude mice and administered gemcitabine or NSC348884 via intraperitoneal injection every other day. NSC348884 treatment attenuated the growth of CSN6‐OE tumors and enhanced gemcitabine efficacy without overt systemic toxicity (Figure 6C and Figure S7B, Supporting Information). Mice receiving the combination therapy exhibited pronounced suppression of tumor growth, volume, and weight (Figure 6C). qPCR analysis of CDXs after drug administration showed that gemcitabine monotherapy promoted rRNA synthesis, indicating a compensatory adaptive response. However, NSC348884 counteracted this GEM‐induced increase in rRNA (Figure 6D). We propose that the upregulation of rRNAs following GEM treatment reflects an adaptive advantage conferred by CSN6 OE, which enables tumors to withstand therapeutic stress and activate resistance mechanisms (including enhanced ribosome biogenesis) during the one‐month sustained treatment period.

IHC staining confirmed OE of CSN6 and decreased expression of DCAF1, which is due to CSN6‐mediated DCAF1 protein degradation. The combination of gemcitabine and NSC348884 resulted in a more significant reduction in the expression of UBTF1, NPM1, and Ki67 (Figure 6E). Notably, this combination treatment also elicited the highest level of γ‐H2AX, suggesting that NSC348884 potentiates the DNA damage effect of gemcitabine in CSN6‐overexpressing tumors. Thus, the NPM1 blockade could be a strategic rationale to re‐sensitize gemcitabine‐resistant PDAC tumors. In conclusion, these data demonstrate that gemcitabine‐NSC348884 combination suppresses the CSN6‐NPM1 regulatory axis and may serve as a promising therapeutic strategy for PDAC patients with high CSN6 expression.

### 2.7 High Expression of CSN6 and NPM1 Predicts Poor Prognosis of PDAC

To evaluate the clinical relevance of CSN6‐mediated NPM1 stabilization in PDAC, IHC staining was conducted on PDAC tissue microarray. Correlation analysis revealed a significant positive association between CSN6 and NPM1 protein expression, as quantified by H‐score evaluation. This clinical observation is consistent with our biochemical findings that CSN6 regulates NPM1 protein stability (Figure 6F). Next, patients were categorized based on CSN6 and NPM1 expression levels using ROC‐derived cutoff values (Figure 6G and Table S2, Supporting Information). Kaplan–Meier survival analysis indicated that patients exhibiting concurrent high expression levels of both CSN6 and NPM1 had significantly shorter overall survival than those with low expression of both proteins, with median survival time reduced from 28 to 6.5 months (Figure 6H). Together, these results demonstrate that CSN6 and NPM1 are co‐expressed in PDAC and that their high co‐expression is associated with poor prognosis.

## 3 Discussion

**Figure 7:**
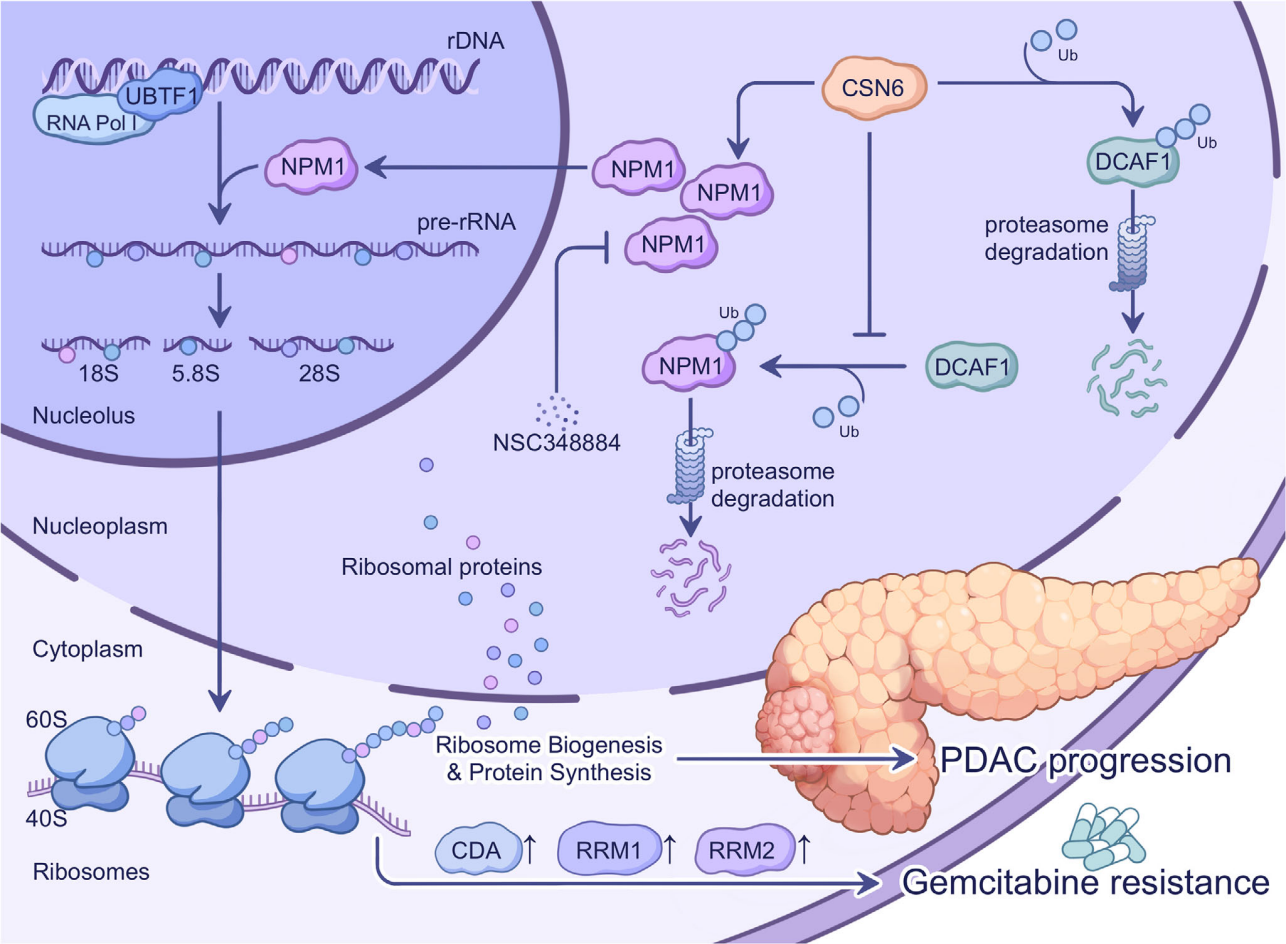
Schematic model of the CSN6‐NPM1 axis in promoting pancreatic cancer progression and gemcitabine resistance. CSN6 promotes the auto‐ubiquitination and degradation of DCAF1, thereby antagonizing DCAF1‐mediated ubiquitination of NPM1. This stabilization of NPM1 enhances the ribosome biogenesis process and the translation of specific gemcitabine‐resistance proteins, ultimately driving tumor progression and chemoresistance. Conversely, pharmacological inhibition of the CSN6‐NPM1 axis suppresses tumor growth and alleviates gemcitabine resistance.

CSN6 is associated with malignant phenotypes in various types of cancer. However, its oncogenic role in PDAC remains largely unknown. Here, we reveal that CSN6 is significantly overexpressed in PDAC and positively correlates with ribosome biogenesis. Proteomic analysis revealed that CSN6 KD led to decreased ribosome biogenesis. CSN6 directly enhances the ubiquitination and destabilization of DCAF1, promoting NPM1 stabilization and NPM1‐associated PDAC ribosome biogenesis. Furthermore, gemcitabine resistance can elevate CSN6 expression, thereby promoting ribosome biogenesis and translation of gemcitabine resistance proteins (CDA/RRM1/RRM2). Targeting the CSN6‐NPM1 signaling axis by combined‐treatment with gemcitabine and an NPM1 inhibitor (NSC348884) improves the therapeutic efficacy of gemcitabine in CSN6‐high PDAC. Collectively, elevated CSN6 expression promotes DCAF1 auto‐ubiquitination, attenuating DCAF1‐mediated NPM1 ubiquitination. The consequent stabilization of NPM1 enhances ribosome biogenesis, driving pancreatic cancer progression and gemcitabine resistance (**Figure** **7**).

We demonstrated that human PDAC tumors expressing high levels of CSN6 present with a poor prognosis. Animal models are critical for better understanding the role of CSN6 in pancreatic cancer. These models are designed to uncover potential therapeutic targets. The KPP and KPPC mice mimicked the natural process of PDAC tumorigenesis and development. The results indicate that *Csn6* KO in pancreatic tissue diminishes tumor formation, suggesting that CSN6 plays a critical role in PDAC development. Although the transgenic cohort was limited, the complete lack of tumor formation in *Csn6*‐KO KPPC mice (as evidenced by 0/3 versus 3/3 in KPP controls) robustly supports a tumor‐suppressive role of CSN6 ablation, which is consistent with our in vitro and xenograft models and aligns with previous findings in other cancer types.^[^ ^11^, ^13^ ^]^

RNA polymerase I (Pol I) mediates the transcription of 5.8S, 18S, and 28S rRNA in the nucleolus, while Pol III mediates the transcription of 5S rRNA outside the nucleolus.^[^ ^35^ ^]^ CSN6 KD leads to downregulation of 18S rRNA, 5.8S, and 28S rRNAs, suggesting that CSN6 is positively involved in ribosome biogenesis, which is consistent with its role in promoting cancer cell growth as cancer cells have a strengthened rRNA transcriptional activity. Furthermore, cancer cells are actively proliferating, thus requiring vigorous ribosomal biosynthesis, and they consume up to 80% of intracellular energy supply.^[^ ^35^ ^]^ Thus, CSN6's role in ribosome biogenesis opens another avenue for understanding its oncogenic role. Previous studies have documented that a subunit of the COP9 signalosome, CSN7b, is involved in ribosome biogenesis although the detailed mechanism remains largely unknown.^[^ ^36^ ^]^ We demonstrate that the CSN6 subunit, structurally different from CSN7b, can enhance ribosome biogenesis via regulating NPM1 stability. NPM1 is highly expressed in proliferating cells^[^ ^37^ ^]^ and various solid tumors and promotes tumor development, consistent with CSN6's oncogenic role. NPM1 is the most frequently mutated protein in acute myeloid leukemia (AML), accounting for approximately one‐third of cases.^[^ ^38^ ^]^ The bipartite NLS is critical for the nuclear import of wt NPM1. The C‐terminal three‐helix NoLS is important for the nucleolar localization of wt NPM1, while the two weak nuclear export signals located at the N terminus mediate NPM1 export from the nucleus to the cytoplasm. CSN6 OE leads to strong accumulation of NPM1 in the nucleolus, presumably enhancing ribosome biogenesis. NPM1 can bind to nascent rRNA and RPs. For example, NPM1 binds RPL23 and is involved in controlling cell proliferation.^[^ ^39^ ^]^ Therefore, by stabilizing NPM1, CSN6 enhances NPM1's role in orchestrating ribosome biogenesis, ultimately promoting protein translation.

CSN6 has been shown to regulate several oncogenic pathways by modulating protein stability through the ubiquitin‐proteasome system, including FASN in lipid metabolism and HMGCS1 in the mevalonate pathway. Our study has added two potential targets: destabilizing DCAF1 yet stabilizing NPM1. DDB1‐Cul4‐associated factor 1 (DCAF1) is a substrate receptor for the DDB1‐CUL4‐ROC1 E3 ubiquitin ligase involved in development, ribosome biogenesis, and cell proliferation. As for ribosome biogenesis, CRL4‐DCAF1 is the ligase for the ribosome assembly factor PWP1. DCAF1 loss leads to PWP1 accumulation, thereby mitigating rRNA processing and ribosome biogenesis.^[^ ^40^ ^]^ As for cancer, DCAF1 is oncogenic in breast cancer, as it negatively regulates BARD1, a tumor suppressor.^[^ ^41^ ^]^ DCAF1 is overexpressed in colon cancer and can phosphorylate histone H2 at T120 to cause oncogenic transformation.^[^ ^42^ ^]^ DCAF1 can phosphorylate p53 at S367, thereby attenuating p53 transcriptional activation and tumor suppressive activities.^[^ ^43^ ^]^ The tumor‐suppressive role of DCAF1 in PDAC appears contradictory to its oncogenic role in other cancer types with respect to the regulation of ribosome biogenesis or oncogenic activity. It is not clear how this discrepancy arises, as DCAF1 is obviously destabilized via oncogenic CSN6‐initiated self‐ubiquitination.

As NPM1 is involved in ribosome biogenesis, it is possible that altered translation may contribute to drug resistance. Indeed, three proteins involved in gemcitabine drug resistance have been characterized in our CSN6‐NPM1 axis. For example, CDA, an enzyme of the pyrimidine salvage pathway, is involved in deaminating gemcitabine, thereby altering transporter affinity and causing gemcitabine drug resistance.^[^ ^44^, ^45^ ^]^ Ribonucleotide reductase large subunit M1 (RRM1), a rate‐limiting enzyme of the DNA synthesis pathway, is involved in converting ribonucleotides to dNTPs.^[^ ^46^ ^]^ It is activated and is related to gemcitabine drug resistance. Ribonucleotide reductase subunit M2 (RRM2), an enzyme that catalyzes the synthesis of deoxyribonucleotides, is involved in tumor progression in many types of cancer, especially upregulated in sunitinib‐resistant RCC cells.^[^ ^47^ ^]^ Studies in *Saccharomyces cerevisiae* indicate that the CSN6 contributes to gemcitabine resistance.^[^ ^48^ ^]^ Data mining of Cancer Therapeutics Response Portal (CTRP) dataset and CCLE indicated that CSN6 expression level indeed correlates with gemcitabine's IC_50_ value in PDAC cell lines. Our data also showed that CSN6 OE promotes polysome formation of RNA (thereby increasing protein synthesis) and engenders gemcitabine resistance in PDAC cells, by enhancing the translation of CDA and RRM1/2.

Given that the activation of CSN6‐NPM1‐ribosome biogenesis axis, at least in part, is the molecular mechanism underlying resistance in gemcitabine‐treated PDAC cells. It is possible that the NPM1 blockade is a strategic rationale to resensitize gemcitabine resistant PDAC tumors. Indeed, NPM1 inhibitor NSC348884 attenuates NPM1‐mediated ribosome biogenesis establishment, i.e., 5.8S‐28S rRNA synthesis, in CSN6‐expressing PDAC receiving gemcitabine therapy. NSC348884 demonstrates strong efficacy in tumor inhibition and gemcitabine sensitization. It was shown that NPM1 inhibition leads to apoptosis and sensitizes NPM1c+‐expressing AML cells to drugs such as ATRA and cytarabine,^[^ ^30^ ^]^ suggesting that NPM1 is a target for cancer therapy. Indeed, in our animal model study, both CSN6 KD (leading to NPM1 decrease) and the NPM1 inhibitor NSC348884 showed strong efficacy in tumor inhibition and gemcitabine sensitization. Another option could be to use CX‐5461, which was developed as a Pol I–specific inhibitor to inhibit the rRNA transcription^[^ ^35^ ^]^ and has been shown to sensitize cells to gemcitabine.

In conclusion, our results demonstrate a CSN6‐NPM1 link in PDAC progression. The effect of CSN6 in enhancing NPM1 stability/ribosome biogenesis via antagonizing DCAF1‐mediated ubiquitination of NPM1 elucidates a new and specific layer of regulation regarding the gemcitabine resistance involved in PDAC progression. Treatment strategies that sensitize gemcitabine via inhibition of NPM1 oligomerization activity by NSC348884 can be further employed as a rational cancer therapy for CSN6‐high PDAC, especially in gemcitabine‐resistant patients.

## 4 Experimental Section

### Tissue Microarray (TMA)

The paraffin‐embedded human pancreatic cancer tissue microarray HPanA180Su10 was obtained from Outdo Biotech (Shanghai Outdo Biotech Co., Ltd.). The use of this tissue microarray was approved by the Ethics Committee of Shanghai Outdo Biotech (Ethical Approval No. SHYJS‐CP‐1901011) and complies with ethical regulations. All tissue samples were collected with informed consent from patients. The array initially contained 90 paired cases; the final analysis included 84 pairs after the exclusion of non‐PDAC samples and spots that had suffered tissue loss or became detached. The IHC‐stained slides were scanned using the Aperio VERSA 8 digital pathology scanner (Leica Biosystems) and analyzed with Aperio ImageScope software and integrated Aperio Image Analysis algorithms (Leica Biosystems).

### Immunohistochemistry (IHC)

Immunohistochemistry was performed following a standard protocol as follows: paraffin‐embedded xenograft tumor tissue sections were deparaffinized in xylene and then hydrated in graded ethanol, followed by antigen repairing in EDTA buffer (ZSGB‐BIO, ZLI‐9069). Then the sections were immersed in 3% hydrogen peroxide to quench endogenous peroxidase activity and then blocked with goat serum (ZSGB‐BIO, ZLI‐9056) for 1 h, followed by incubation with primary antibodies at 4 °C overnight. On the next day, sections were incubated with secondary antibodies (anti‐rabbit/mouse IgG; ZSGB‐BIO, PV‐6000) at room temperature for 30 min, and then the staining was visualized using diaminobenzidine (DAB) (ZSGB‐BIO, ZLI‐9018), followed by hematoxylin (Servicebio, G1004) staining for 3 min and then differentiated in hydrochloric alcohol. After air‐drying, the sections were mounted with neutral resin under a cover slip. ImageJ was used for IHC quantification.

### Cell Culture and Transfection

All pancreatic cancer cell lines and HEK293T cells were obtained from American Type Culture Collection (ATCC), confirmed to be mycoplasma‐free, and cultured in a cell culture incubator at 37 °C with 5% (vol/vol) CO_2_. The RRIDs of these cell lines are listed as follows: HEK293T: CVCL_0063; PANC‐1: CVCL_0480; MiaPaCa‐2: CVCL_0428; BXPC‐3: CVCL_0186. Cells were cultured in Dulbecco's modified Eagle's medium (DMEM) (Corning) and/or RPMI 1640 medium (Corning) supplemented with 10% FBS. Transient transfection: polyethylenimine (PEI) (Polysciences, 24 765) was used for cell transfection in this study following the manufacturer's standard protocol.

### Plasmid Construction

The cDNA of NPM1 (NM_0 02520.7) was amplified by PCR and then cloned into pcDNA3.1‐Flag using the ClonExpressII One Step Cloning Kit (Vazyme) according to the manufacturer's protocol. pcDNA3‐HA‐DCAF1 (NM_01 4703) was obtained from Addgene. CSN6 (NM_0 06833.5) OE constructs and other plasmids were previously constructed in our laboratory.^[^ ^10^ ^]^ The shRNAs were cloned into pLKO‐Tet‐On vector or pLKO.1 vector. The targeting sequences of the shRNAs are listed in Table S3 (Supporting Information). All plasmids and constructs were verified by DNA sequencing.

### Lentivirus Production and RNA Interference

HEK293T cells were seeded and then co‐transfected with shRNA plasmid, psPAX2 and pMD2.G by PEI. The supernatant was collected at 48 and 72 h and then filtered through a Millex‐GP filter unit (Millipore, 0.45 µm pore size). Cells were infected with the lentivirus‐containing supernatant supplemented with 10 µg mL^−1^ polybrene (Millipore, TR‐1003‐G) and then selected with puromycin. For pLKO‐Tet‐On system, 200 ng mL^−1^ doxycycline (Selleck, S4163) was added to the culture medium to induce KD of the target genes. Lentivirus‐mediated RNA interference and target gene KD were validated by qPCR and/or Western blotting prior to their use in subsequent experiments.

### Cell Proliferation and Viability Analysis

Cell proliferation and viability were examined using the Cell Counting Kit‐8 (CCK‐8) assay (APExBIO). A 10% (v/v) CCK‐8 solution in culture medium was added to each well and incubated for 3 h. Then the absorbance (OD) was measured at a wavelength of 450 nm using a microplate reader.

### Foci Formation

Cells were seeded and cultured at 37 °C with 5% CO_2_ for 7 days. Clones were fixed with 4% paraformaldehyde for 10 min, followed by staining with 0.5% crystal violet for 20 min, washing, and imaging. Quantification was performed using ImageJ. For each sample, three biological replicates were performed in each experiment.

### Xenograft PDAC Model in Nude Mice

These experiments were approved by the Animal Ethical and Welfare Committee of the Sixth Affiliated Hospital of Sun Yat‐sen University (Ethical code: IACUC‐2021071201). Cells were pretreated and inoculated subcutaneously into the right hind flanks of 4‐ to 6‐week‐old BALB/c‐nu/nu mice. For the CSN6 and gemcitabine treatment experiment, mice were i.p. injections with one of the following: saline, 50 mg kg^−1^ gemcitabine (Aladdin, 122111‐03‐9), 50 mg kg^−1^ doxycycline (Selleck, S4163), or a combination of 50 mg kg^−1^ gemcitabine plus 50 mg kg^−1^ doxycycline every other day. For the NSC348884 and gemcitabine experiment, mice were divided into four groups and treated every other day by i.p. injection with one of the following: saline, 50 mg kg^−1^ gemcitabine, 5 mg kg^−1^ NSC348884 (Aladdin, 81624‐55‐7),^[^ ^49^ ^]^ and 50 mg kg^−1^ gemcitabine plus 5 mg kg^−1^ NSC348884. Both doxycycline and gemcitabine were dissolved in saline, while NSC348884 was dissolved in a vehicle consisting of 2% DMSO, 50% PEG300, 0.1% Tween‐80, and 47.9% saline. For NSC348884 and gemcitabine experiment, body weight was monitored to assess potential toxicity. At the experimental endpoint, mice were euthanized by CO_2_ inhalation, and tumors were harvested for further analysis. All BALB/c‐nu/nu mice were purchased from Guangdong GemPharmatech Co., Ltd. (Guangdong, China), and housed under specific pathogen‐free (SPF) conditions in the Laboratory Animal Center of the Sixth Affiliated Hospital, Sun Yat‐sen University.

### KPP and KPPC Mouse Models

This experiment was approved by the Animal Ethical and Welfare Committee of the Sixth Affiliated Hospital of Sun Yat‐sen University (Ethical code: IACUC‐2021071201). *Csn6^fl/fl^* (Cops6‐flox mice, Strain NO. T008614), *Kras* ^G12D^ *Trp53* ^R172H^ (B6‐KP mice, Strain NO. T015831), and *Ptf1a* ^CreERT2^ (Ptf1a‐P2A‐CreERT2 mice, Strain NO. T006208) mice on a C57BL/6J background were generated by CRISPR/Cas9 knock‐in or targeted mutation methods and were purchased from GemPharmatech (Nanjing, China). KPP and KPPC mice were confirmed by genotyping before tamoxifen induction for further experiments. To induce heterozygous *Kras* plus *Trp53* activation or pancreas‐specific and conditional homozygous KO of *Csn6*, KPP, and KPPC mice at 4–6 weeks of age were injected intraperitoneally with 100 mg kg^−1^ tamoxifen (Aladdin, T137974) for five consecutive days.

### Proteomic Assay and IP‐MS

For the proteomic assay, PANC‐1 cells were infected with sh SC or shCSN6‐1 lentivirus and biological triplicates from these groups were subjected to proteomic analysis by Shanghai Applied Protein Technology Co., Ltd. For IP‐MS, lysates from MiaPaCa‐2 cells were prepared and incubated with an anti‐CSN6 or control IgG antibody, followed by incubation with protein A/G Sepharose beads to immunoprecipitate CSN6‐associated proteins. The immunoprecipitated complexes were then submitted to Shanghai Applied Protein Technology Co., Ltd for MS.

### RNA Extraction, Reverse Transcription, and Quantitative PCR (qPCR)

Total RNA was extracted using Trizol Reagent (Invitrogen, 15 596 026) following the standard RNA extraction protocol. The isolated RNA was reverse transcribed to complementary DNA (cDNA) using ReverTra Ace qPCR RT Master Mix with gDNA Remover (TOYOBO) according to the manufacturer's instructions. Relative gene expression was quantified by quantitative real‐time PCR using 2× SYBR Green qPCR Master Mix (Bimake, B21203) on a LightCycler 480II instrument (Roche). The transcript levels of all target genes were normalized to *β‐actin*. Technical triplicates were performed for each sample. All qPCR primer sequences are listed in Table S4 (Supporting Information).

### Transmission Electron Microscopy (TEM)

Transfected control or CSN6‐OE BXPC‐3 cells were fixed with fixative solution (Servicebio, G1102) for 24 h. After being washed with 0.1 M phosphate buffer, the cells were post‐fixed in 1% osmium tetroxide (Ted Pella Inc) for 2 h at room temperature, followed by dehydration through a graded ethanol series and embedding in SPIPon 812 resin (Structure Probe, Inc.). The embedded blocks were polymerized at 60 °C for 48 h. Ultrathin sections (60–80 nm) were prepared using a Leica Ultracut microtome (Leica UC7) and double‐stained with 2% uranyl acetate and 2.6% lead citrate. Nucleolar ultrastructure was examined using a Hitachi TEM system (HT7800).

### Ribosome Profiling Assay and Translation Efficiency Analysis

For polysome profiling, 1 × 10^7^ BXPC‐3 cells were treated with 100 µg mL^−1^ cycloheximide for 15 min and then lysed with ice‐cold polysome extraction buffer and centrifuged at 13000 × *g* for 10 min at 4 °C. After that, 1 mL of the cytoplasmic extract was layered onto 11 mL 10%–50% sucrose gradient and then centrifuged at 36 000 rpm for 3 h at 4 °C using a SW41 rotor (Beckman Coulter, USA). Separated samples were fractionated at 0.75 mL min^−1^ using a BR‐188 Density Gradient Fractionation System (Brandel, USA) while monitoring absorbance at 254 nm. Fractions corresponding to monosomes and polysomes were collected for RNA isolation to analyze the relative distribution of β‐actin, CDA, RRM1, and RRM2 mRNAs. Total RNA was isolated from each fraction using an RNA‐Quick Purification Kit (ESscience).^[^ ^50^ ^]^

### Western Blot Analysis

Total cell lysates were prepared using ice‐cold lysis buffer. Protein concentrations were quantified and equalized using a BCA protein assay kit (BestBio, 3401). Equal amounts of protein were separated by SDS‐PAGE and subsequently transferred onto a PVDF membrane (Millipore, IPVH00010). The membrane was then incubated with primary antibodies followed by horseradish peroxidase (HRP) conjugated secondary antibodies. Signals were visualized using enhanced chemiluminescence (ECL) reagent (BioRad, 1 705 061) and captured with chemiluminescence imaging systems.

The primary and HRP‐conjugated secondary antibodies used in this study are listed below: anti‐CSN6 (Enzo Life Sciences, BML‐PW8295, 1:2000), anti‐γ‐H2AX (Cell Signaling Technology, 9718s, 1:1000), anti‐α‐Tubulin (Proteintech, 66031‐1‐Ig, 1:4000), anti‐puromycin (Sigma‐Aldrich, MABE343, 1:1000), anti‐Flag‐tag (Sigma, F1804, 1:4000), anti‐NPM1 (ThermoFisher, 325 200, 1:4000), anti‐DCAF1 (Proteintech, 11612‐1‐AP, 1:4000), anti‐Myc‐tag (Cell Signaling Technology, 2276s, 1:4000), anti‐HA‐tag (Proteintech, 51064‐2‐AP, 1:2000), and anti‐UBTF1 (Santa Cruz, sc‐13125, 1:1000).

### Puromycin Incorporation Assay

Cells were cultured 48 h before replacing the medium with fresh cell culture medium containing 10 µg mL ^−1^ puromycin and incubating in a cell culture incubator for 30 min. Cells were harvested for further Western blot analysis with an anti‐puromycin antibody.

### Immunoprecipitation

For the in vivo IP, cell lysates were prepared as mentioned before and rotated with appropriate primary antibodies or anti‐Flag M2 beads (Sigma‐Aldrich, F1804) at 4 °C overnight. Protein A/G Sepharose (Santa Cruz, sc2003) was added if the primary antibody was used for endogenous immunoprecipitation. After washing the beads five times with lysis buffer, the beads were then boiled for further SDS‐PAGE and Western blot analysis. For the in vitro IP, Quick Coupled Transcription/Translation systems (Promega, no. L1170) were employed to generate targeted proteins in vitro, following the standard protocols. Specific proteins were pulled down using anti‐Flag M2 agarose beads, followed by immunoblotting with indicated antibodies.^[^ ^51^ ^]^

### Molecular Docking

The protein–protein interactions were predicted using AlphaFold3. The predicted structures were analyzed with Pymol v2.3.4 to obtain the amino acid pairs that can form hydrogen bonds between proteins.

### Turnover Rate Assay

Cells were seeded and cultured to a confluency of 80%, followed by treatment with 20 µg mL^−1^ cycloheximide (CHX) (Sigma‐Aldrich, C7698). At separate times, cells were harvested and analyzed by Western blot.

### His‐Tagged Ubiquitination Assay

HEK293T or PDAC cells were co‐transfected with His‐ubiquitin vector and the indicated plasmids for 48 h. After treatment with 10 µm MG132 (Selleck, S2619), cells were lysed in denaturing buffer A (6 m guanidine‐HCl, 10 × 10^−3^ m imidazole, 0.1 m Na2HPO4/NaH2PO4 pH 8.0). Then nickel‐nitrilotriacetic acid (Ni‐NTA) agarose beads (Invitrogen, R901‐15) were added to the cell lysate samples and incubated at 4 °C overnight. On the next day, Ni‐NTA agarose beads were washed with Washing Buffer (25 × 10^−3^ m Tris‐HCl, 20 × 10^−3^ m Imidazole) and boiled in SDS loading buffer. The target proteins were eluted and prepared for Western blot analysis.^[^ ^52^ ^]^

### Immunofluorescence (IF)

Cells were seeded on the bottom glass in cell culture dish (NEST, 801 002) and cultured to the ideal density. After washing with PBS, cells were fixed with 4% paraformaldehyde for 10 min at room temperature, then permeabilized with 0.5% Triton X‐100 for 10 min. Subsequently, cells were blocked with 2% bovine serum albumin for 1 h at room temperature and then incubated with the desired primary antibodies at 4 °C overnight. On the next day, samples were incubated with Alexa Fluor 594‐conjugated secondary antibody (Invitrogen, A‐11005, 1:1000) for 1 h, and then the cell nuclei were stained with DAPI (Invitrogen, 62 248, 1:10 000). Fluorescence signals were detected and imaged by Leica TCS SP8 Laser Scanning Confocal Microscope.

### Chromatin Immunoprecipitation (ChIP)

ChIP was performed following previously described protocols.^[^ ^51^ ^]^ Control, CSN6‐OE, NPM1‐KD and CSN6‐OE plus NPM1‐KD MiaPaCa‐2 cells were cross‐linked with 1% formaldehyde for 10 min at 37 °C, quenched with glycine, and washed with cold PBS. After scraping and centrifugation, cells were lysed in ChIP lysis buffer with protease inhibitors and sonicated using Diagenode Bioruptor Pico sonicator. Chromatin lysates were incubated overnight at 4 °C with 20 µL of Magna ChIP Protein A + G Magnetic Beads (Millipore, 16‐663) that had been pre‐bound with 2 µg of antibody against UBTF1 (Santa Cruz, sc‐13125) or IgG. Beads were sequentially washed with low‐salt, high‐salt, LiCl, and TE buffers, then eluted. Eluates were de‐crosslinked overnight with NaCl and Proteinase K (20 mg mL^−1^) at 55 °C. DNA was purified using a PCR purification kit (Omega, D2500‐02) and analyzed by qPCR.

### Apoptosis Assay by Flow Cytometry

Cells were seeded and treated with shRNAs or gemcitabine for 48 h. Then, according to the manufacturer's instructions for the Annexin V‐FITC/PI apoptosis kit (Multi Science, 70‐AP101), the cells were collected for analysis by fluorescence‐activated cell sorting (FACS) using CytoFLEX (Beckman Coulter, A00‐1‐1102). The results were analyzed and presented by FlowJo software.

### Bioinformation Analysis

Publicly available PDAC associated datasets were obtained from the Gene Expression Omnibus (GEO), The Cancer Genome Atlas (TCGA), or CPTAC_PDA datasets, and The Cancer Cell Line Encyclopedia (CCLE).

### Statistics

All statistical analyses were performed using GraphPad Prism and/or SPSS 16.0. Kaplan‐Meier survival analyses were used to compare survival among PDAC patients based on CSN6 and/or NPM1 expression; the log‐rank test was used to generate *p* values. Significance was set at *p* < 0.05. Differences between groups were evaluated using Student's t test or one‐way ANOVA. Fisher's exact test (one‐tailed) was used to analyze tumor incidence in a genetically engineered PDAC mouse model. Two‐way ANOVA was used to compare cell growth or tumor growth curves. Spearman's rank correlation was used to analyze the expression correlation between proteins or mRNA. All the data were presented as means ± SD. Significance in the figures was indicated as follows: ns for *p* > 0.05; ^*^ *p* < 0.05; ^**^ *p* < 0.01; and ^***^ *p* < 0.001.

## Conflict of Interest

The authors declare no conflict of interest.

## Author Contributions

M.‐H.L. conceived the research. M.‐H.L. and Y.Z. designed the experiments. Y.Z. conducted bioinformatic analyses. Y.Z., H.L., Z.F. contributed to xenograft experiments. Y.Z. and H.L. performed mouse breeding. Y.Z. performed the cell experiment and IHC staining. Y.Z. performed most of the biochemical and molecular experiments with assistance from H.G., A.T., Y.R., Y.W., and J.G.; Q.L. helped with nascent protein synthesis assessment. Q.L., X.M., H.G., J.G., Q.P., H.L., B.Z., B.Q. contributed to discussion and data interpretation. Y.Z. and M.‐H. L. wrote the manuscript.

## Ethics Approval Statement

The animal experiments were approved by the Animal Ethical and Welfare Committee of the Sixth Affiliated Hospital of Sun Yat‐sen University (Ethical code: IACUC‐2021071201).

## Patient Consent Statement

The use of human PDAC tissue microarray was approved by the Ethics Committee of Shanghai Outdo Biotech (Approval No. SHYJS‐CP‐1901011), with written informed consent obtained from all participants.

## Supporting information



Supporting Information

## Data Availability

The data that support the findings of this study are available from the corresponding author upon reasonable request.
