# Supplementary material for: CSN6 Promotes Pancreatic Cancer Progression and Gemcitabine Resistance via Antagonizing DCAF1‐Mediated Ubiquitination of NPM1
Source: Adv Sci (Weinh). 2025 Oct 20;13(1):e10210. doi: 10.1002/advs.202510210 (PMC12767123; doi:10.1002/advs.202510210)
Supplement: Supplementary file 1 — Supporting Information [file ADVS-13-e10210-s001.docx]

Supporting Information

**CSN6 promotes pancreatic cancer progression and** **Gemcitabine resistance via antagonizing DCAF1-mediated ubiquitination of NPM1**

*Yijing Zhang, Han Gao, Aiwen Tang, Haiwen Lyu, Zongmin Fan, Jiahui Guo, Yuzhi Wang, Hairong Yi, Qihao Pan, Haidan Luo, Baifu Qin, Boyu Zhang, Xiangqi Meng, Qingxin Liu, Mong-Hong Lee**

**
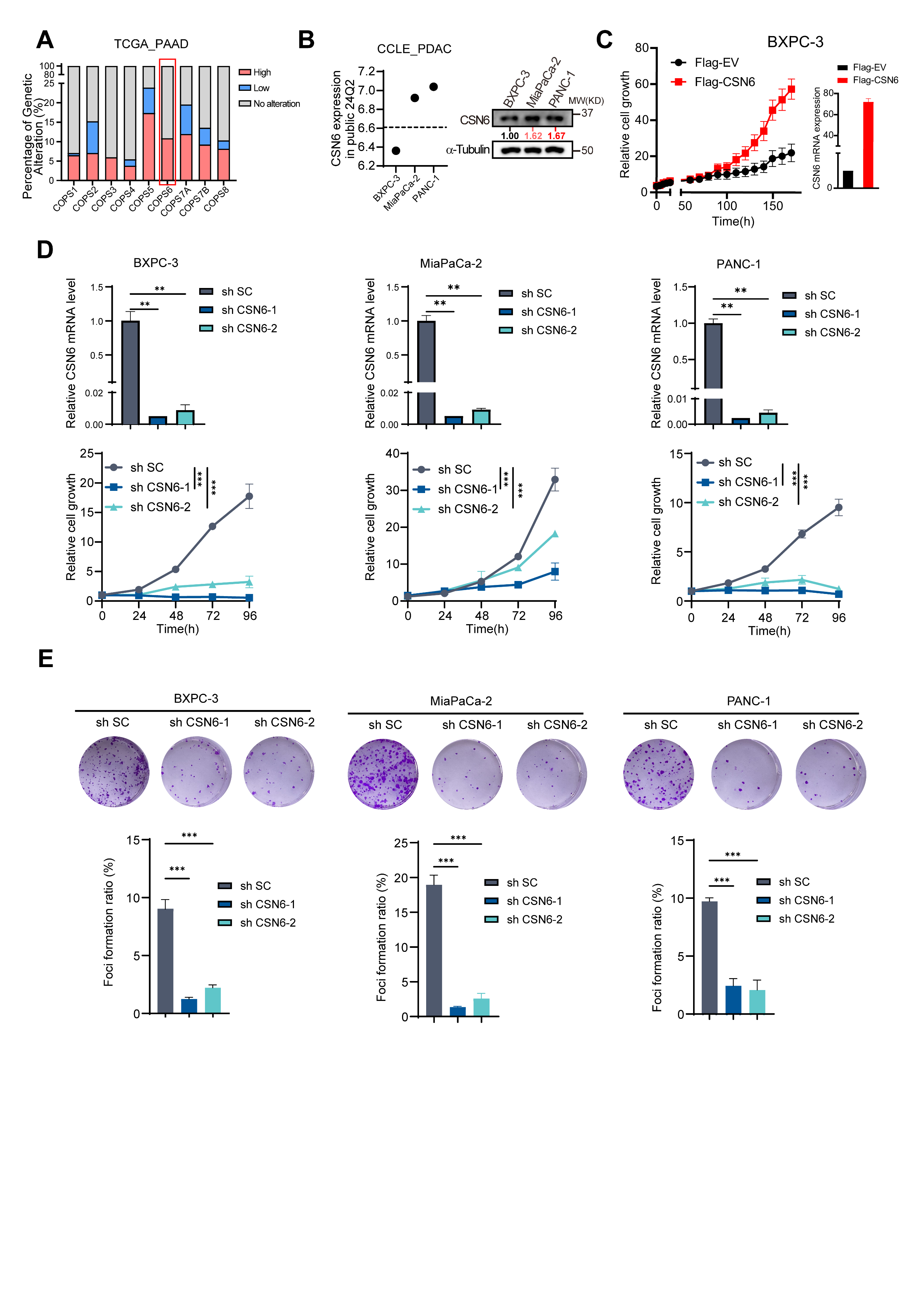
Supplemental Figure 1. CSN6 promotes proliferation of PDAC cells.**

A) Genetic alterations of COP9 signalosome subunits in the TCGA pancreatic cancer dataset.

B) CSN6 mRNA expression levels in PDAC cell lines from the CCLE database (left). The dotted line indicates the mean expression value across 51 cell lines. Immunoblot analysis of CSN6 protein levels in selected cell lines, which is consistent with the CCLE data (right).

C) Proliferation of control and CSN6-OE BXPC-3 cells monitored by IncuCyte (left). Validation of CSN6 overexpression by qPCR (right).

D) CSN6-KD efficiency confirmed by qPCR (top). Proliferation of control and CSN6-KD PDAC cells assessed by CCK-8 assay (bottom).

E) Representative images of foci formation assay (top). Quantification of foci formation in control and CSN6-KD cells (bottom).

Data are presented as mean ± SD. Statistical significance was determined by two-way ANOVA (C, D-proliferation) or one-way ANOVA (D- mRNA level, E). Significance is denoted as ***p* < 0.01, ****p* < 0.001.


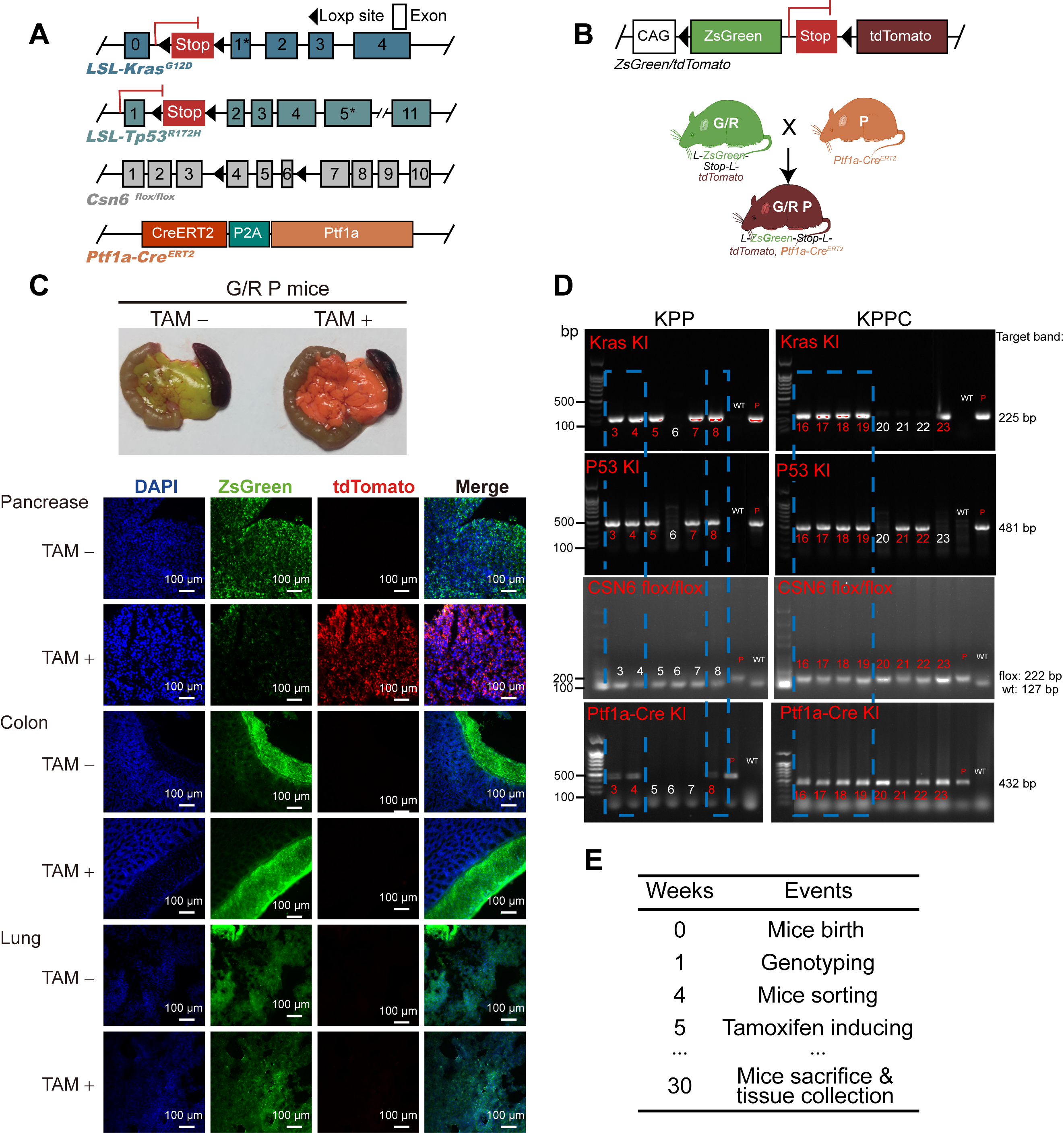
Supplemental Figure 2. Pancreas-specific Cre recombinase activity and genotyping of KPP/KPPC mice.

A) Schematic of *LSL-Kras^G12D/+^; LSL-Trp53^R172H/+^; Csn6^flox/flox^* and *Ptf1a-Cre^ERT2^* conditional allele.

B) Structure of the *ZsGreen/tdTomato* (G/R) reporter allele (top). Breeding strategy for generating G/R P mice to monitor Cre activity (bottom).

C) Tamoxifen-induced fluorescence switch (from ZsGreen to tdTomato) in pancreatic tissues of G/R P mice (top). Ex vivo fluorescence imaging confirmed pancreas-specific recombination (tdTomato signal), with no recombination detected in colon or lung tissues (bottom). Scale bars: 100 μm. Mice received tamoxifen (100 mg kg^-1^, *i.p.*) at 5 weeks of age, and tissues were harvested one week later.

D) Genotyping PCR for identifying KPP (left) and KPPC (right) mice. PCR primers detected: *Kras ^G12D^* knock-in (KI) (first row), *Trp53 ^R172H^* KI (second row), *Csn6* wild-type or flox alleles (third row), and *Ptf1a-Cre^ERT2^* KI (fourth row). Target bands and their sizes are indicated. Blue dashed circles highlight the genotypes of KPP and KPPC mice.

E) Workflow of tamoxifen-induced activation of oncogenic mutations and simultaneous ablation of *Csn6*.


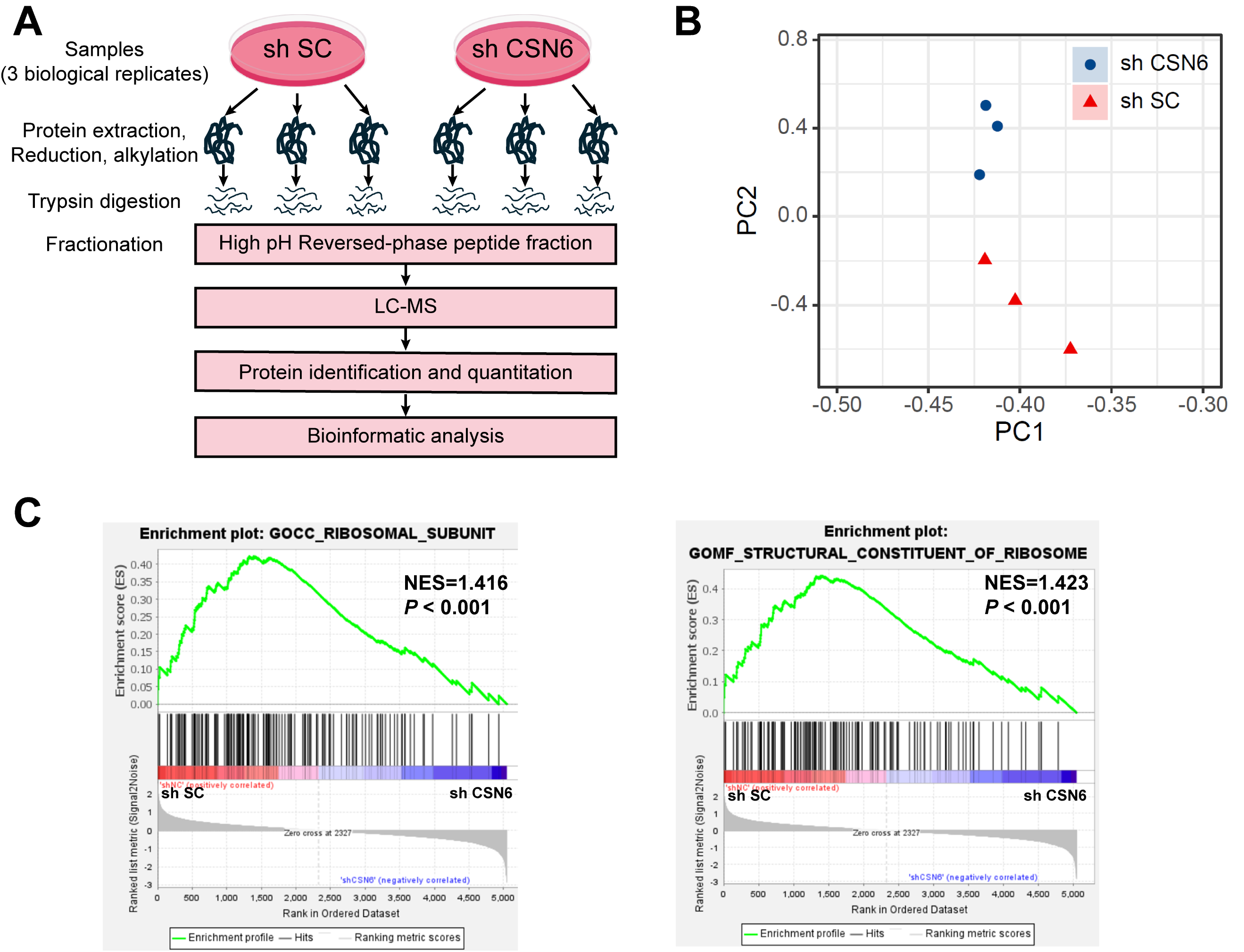
**Supplemental Figure 3. Proteomic analysis suggests a potential role of CSN6 in ribosome biogenesis.**

A) Proteomic workflow and sample groups.

B) PCA shows that CSN6 perturbation markedly reorganizes global protein expression profiles.

C) Proteomic GSEA indicates that ribosomal subunit and ribosome structural constituent terms are enriched in the shSC group compared to the shCSN6 group.

**
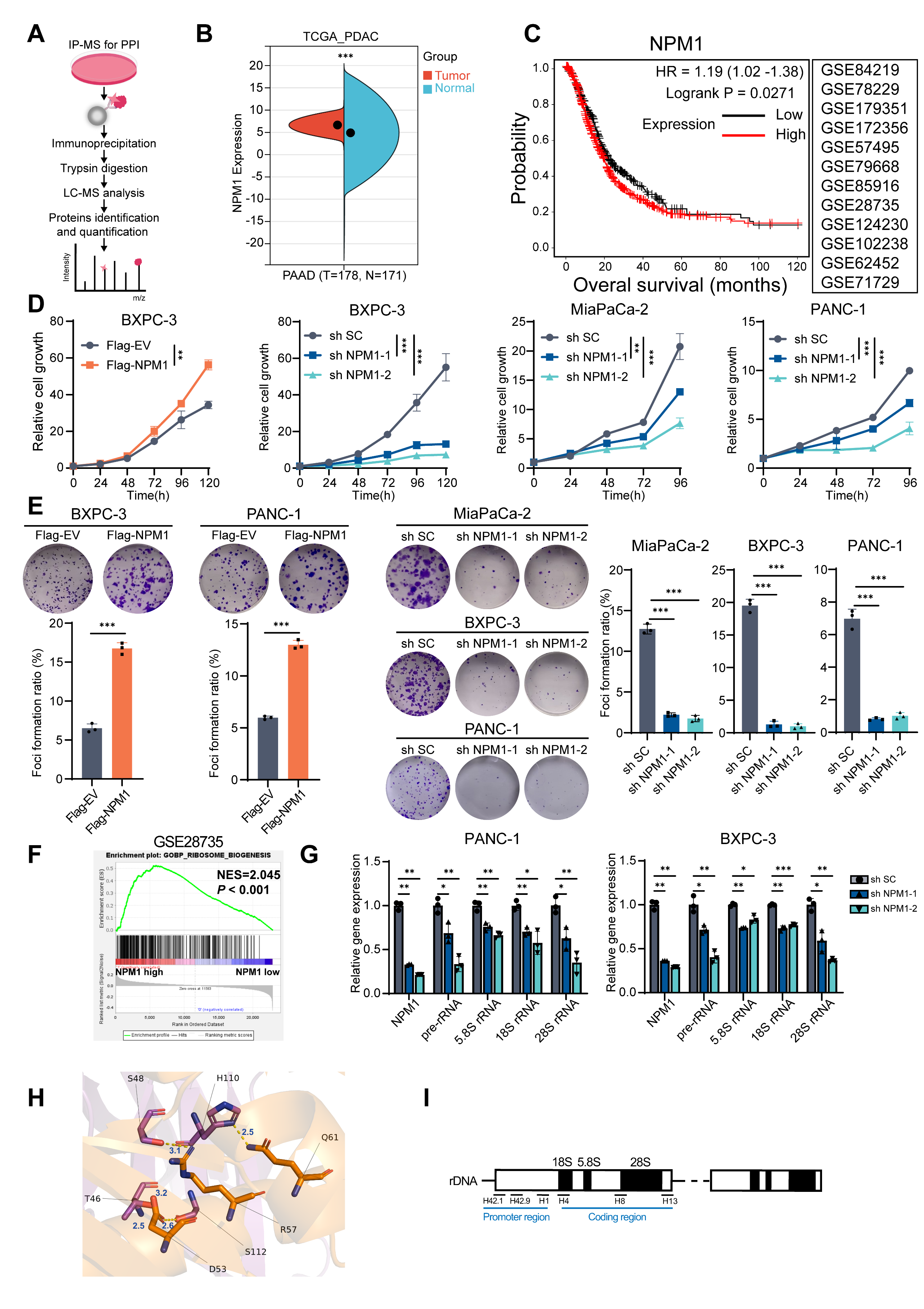
Supplemental Figure 4. CSN6-driven NPM1 expression promotes ribosome biogenesis and PDAC progression.**

A) Schematic workflow of immunoprecipitation-mass spectrometry (IP-MS).

B) Elevated NPM1 expression in TCGA pancreatic tumor vs. normal tissues.

C) Kaplan-Meier survival analysis of PDAC patients stratified by NPM1 expression across 12 PDAC datasets.

D) CCK-8 assay evaluating PDAC cell proliferation capacity upon NPM1-OE or NPM1-KD.

E) Foci formation and quantification assessing PDAC cells’ proliferation ability upon NPM1-OE (left) and knockdown (right).

F) GSEA of GSE28735 stratified by NPM1 expression revealed enrichment of the ribosome biogenesis pathway in the NPM1-high group.

G) qPCR confirming NPM1-KD reducing rRNA synthesis in PDAC cells.

H) Computational modeling of CSN6-NPM1 interactions by AlphaFold3 followed by PyMOL v2.5 visualization. Hydrogen bond distances between interacting residues are annotated in blue. Proteins color assignment: CSN6 (orange), NPM1 (purple).

I) Schematic representation of UBTF1’s binding regions on rDNA.

Data are presented as the mean ± SD. Statistical analysis was performed using Student’s t-test (B, E-NPM1-OE), log-rank test (C), two-way ANOVA (D), or one-way ANOVA (E-NPM1-KD, G). Statistical significance in the figures is indicated as follows: **p* < 0.05, ***p* < 0.01, ****p* < 0.001.

**
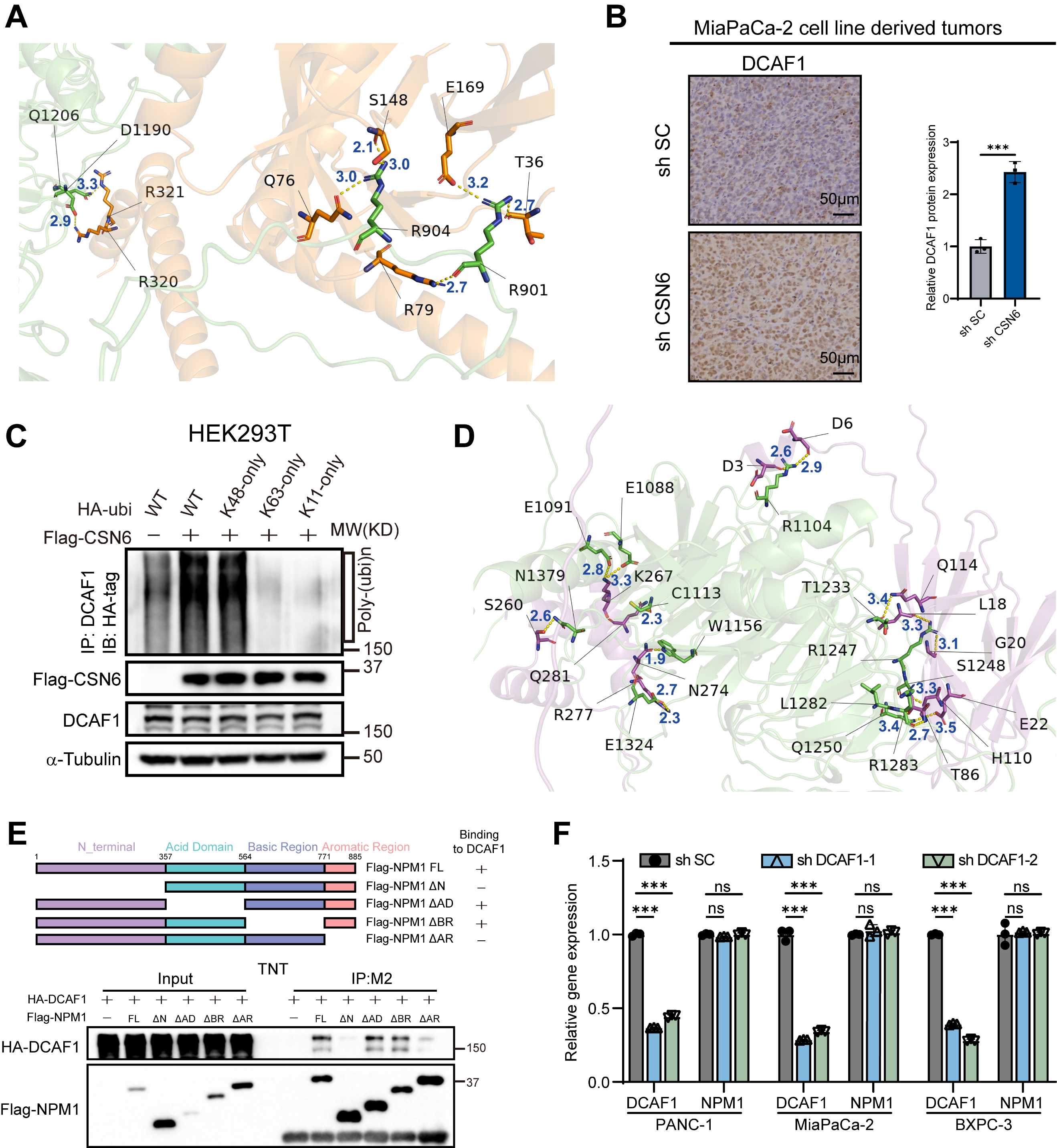
Supplemental Figure 5. CSN6 mediates K48-linked ubiquitination of DCAF1 to post-transcriptionally regulate NPM1.**

A) Computational modeling of CSN6-DCAF1 interactions was performed by AlphaFold3 and visualized with PyMOL v2.5. Hydrogen bond distances between interacting residues are shown in blue. Color assignment for proteins: CSN6 (orange), DCAF1 (green).

B) Representative IHC staining of DCAF1 (left) and ImageJ quantification (right) in MiaPaCa-2-derived xenograft tumors.

C) HA-tagged single-lysine-remained ubiquitin mutant study revealed that CSN6-OE mediated DCAF1 ubiquitination through K48 linkage. HEK293T cells were transfected as indicated and treated with MG132 (10 μm, 6 h), followed by DCAF1 antibody immunoprecipitation and anti-HA immunoblotting.

D) Computational modeling of DCAF1-NPM1 interactions was performed by AlphaFold3 and visualized with PyMOL v2.5. Hydrogen bond distances between interacting residues are shown in blue. Color assignment for proteins: NPM1 (purple), DCAF1 (green).

E) Schematic depiction of full-length (FL) and truncation constructs of NPM1(top). HA-DCAF1 and Flag-NPM1 were co-expressed in vitro using the TNT Quick Coupled Transcription/Translation system, followed by pull-down with anti-Flag M2 beads and immunoblotting with an anti-HA antibody (bottom).

F) DCAF1 knockdown had no impact on NPM1 mRNA expression levels.

Data are presented as the mean ± SD. Statistical analysis was performed using Student’s t-test (B) or one-way ANOVA (F). Statistical significance is indicated as follows: ns for *p* > 0.05, ****p* < 0.001.

**
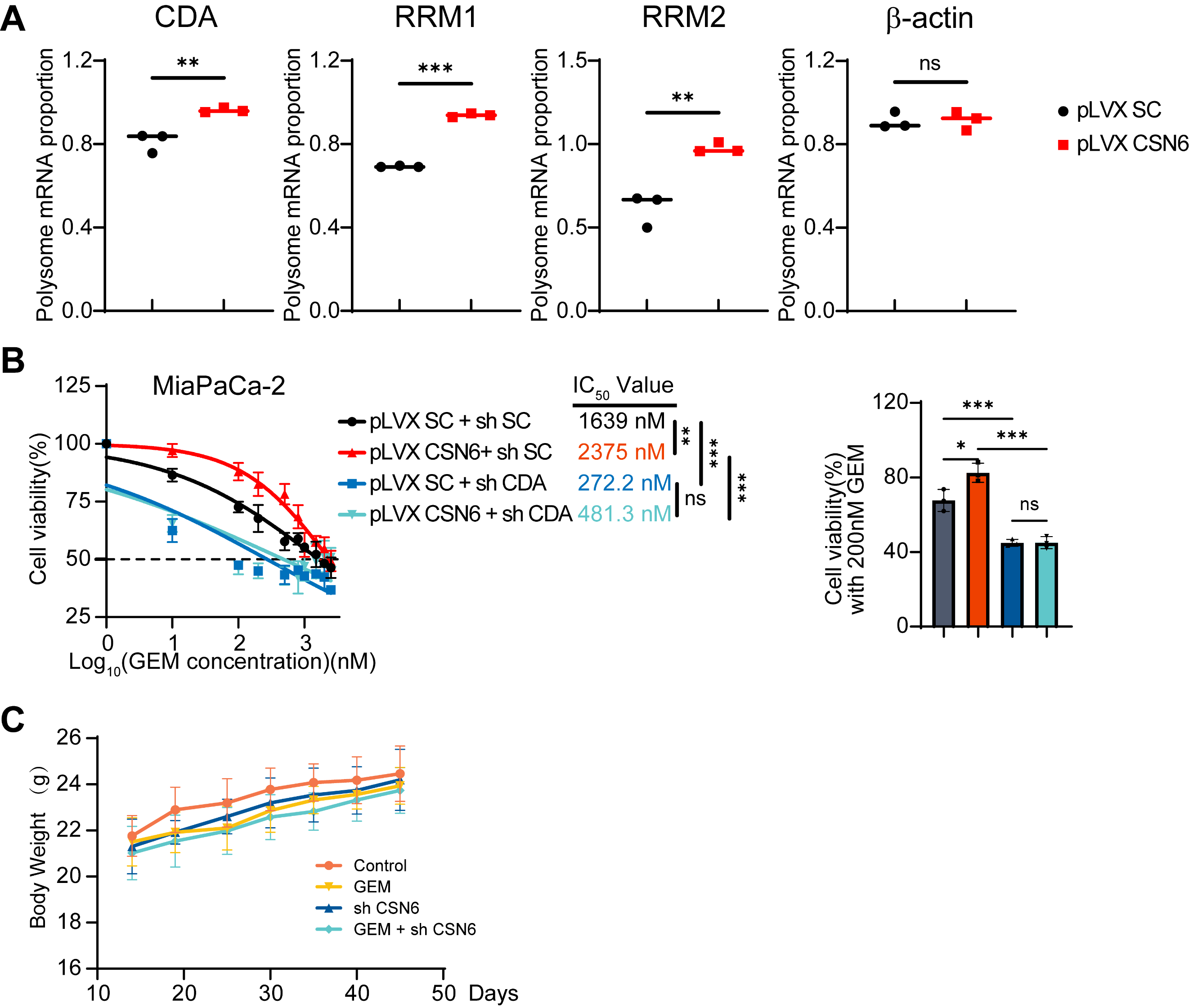
Supplemental Figure 6. CSN6 increases the translation of drug-resistance proteins, leading to gemcitabine resistance.**

A) Ribosome profiling-based qPCR revealed that translation of the indicated proteins was activated by CSN6-OE. Ribosome profiling fractions were divided into monosomal and polysomal fractions and analyzed by qPCR (β-actin as the negative control).

B) Knockdown of gemcitabine resistance-associated gene CDA reversed CSN6 overexpression-induced chemoresistance. IC_50_ was compared by nonlinear regression with labeled values.

C) Body weight monitoring during indicated drug administration.

Data are presented as the mean ± SD. Statistical analysis was performed using Student’s t-test (A), or nonlinear regression for IC50 comparison (B- IC50 dose-response curve), or one-way ANOVA (B-bar graph). Statistical significance in the figures is indicated as follows: ns for *p* > 0.05, ***p* < 0.01, ****p* < 0.001.

**
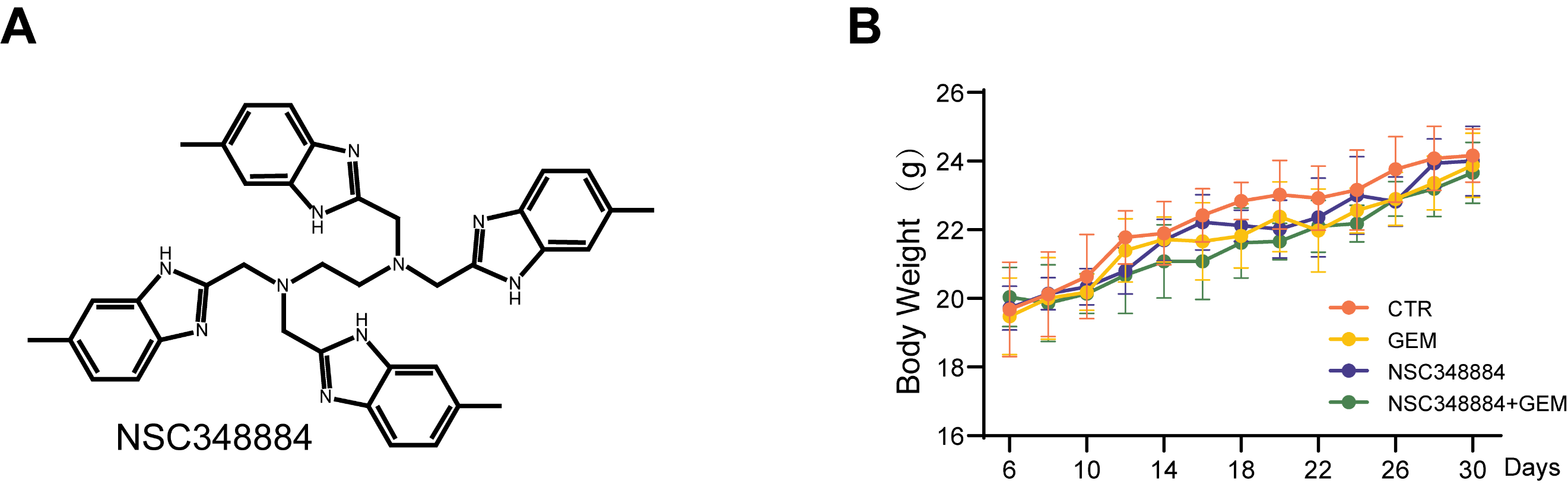
Supplemental Figure 7. The NPM1 inhibitor NSC348884 was well-tolerated in mice.**

A) Chemical structure of the NPM1 oligomerization inhibitor NSC348884.

B) Body weight monitoring during indicated drug administration.

**Supplemental Table 1. CSN6 interactors in MiaPaCa-2 cells identified by IP-MS**

| Accession ^a)^ | C% ^b)^ | #UP ^C)^ | #P ^d)^ | Abundance ^e)^ |  | Accession | C% | #UP | #P | Abundance |
| --- | --- | --- | --- | --- | --- | --- | --- | --- | --- | --- |
| P35527 | 66 | 29 | 28 | 8292458283 |  | Q9Y4B6 | 6 | 7 | 7 | 48123098.88 |
| H6VRF8 | 61 | 30 | 26 | 9004342004 |  | Q7Z794 | 13 | 7 | 4 | 5335866.875 |
| P35579 | 30 | 51 | 45 | 489592238.6 |  | A0A669KAX4 | 8 | 6 | 6 | 28147029.88 |
| P13645 | 51 | 26 | 22 | 6612904488 |  | P22061 | 25 | 4 | 4 | 96481054 |
| P35908 | 66 | 38 | 29 | 2968164049 |  | P23396 | 45 | 10 | 10 | 114188263.9 |
| B7Z4V2 | 47 | 27 | 27 | 1950097004 |  | P19338 | 12 | 9 | 9 | 49329140.25 |
| Q53FV3 | 60 | 18 | 18 | 1446644608 |  | Q8N1N4 | 14 | 7 | 5 | 23649783.63 |
| P08670 | 61 | 30 | 27 | 3664015648 |  | Q53HQ7 | 17 | 7 | 7 | 115100422.6 |
| B4E3A4 | 48 | 12 | 1 | 11492335 |  | P00450 | 4 | 4 | 4 | 118671817.6 |
| Q53G99 | 45 | 12 | 1 | 844015225.8 |  | B2RDE1 | 32 | 8 | 6 | 32558110.44 |
| Q92905 | 65 | 15 | 15 | 784271369.9 |  | P78386 | 16 | 10 | 4 | 51672567.44 |
| P02533 | 54 | 21 | 8 | 399374321.4 |  | Q9NZT1 | 30 | 3 | 3 | 22628733.75 |
| Q9UNS2 | 48 | 16 | 16 | 1797520430 |  | P02786 | 9 | 6 | 6 | 22201223.5 |
| P61201 | 37 | 14 | 14 | 791989436.8 |  | Q13885 | 12 | 5 | 1 |  |
| P08779 | 51 | 21 | 4 | 1007845379 |  | P62877 | 17 | 1 | 1 | 7515649.5 |
| E7EM64 | 48 | 10 | 10 | 393681524.3 |  | P0DPH8 | 14 | 5 | 5 | 69545983 |
| P02538 | 40 | 24 | 2 | 36110646.25 |  | A0A286YEY4 | 17 | 6 | 3 | 12823072.75 |
| P15924 | 12 | 36 | 36 | 161452880.4 |  | B4DJI2 | 7 | 3 | 3 | 29866819.13 |
| Q9H9Q2 | 41 | 9 | 9 | 635023865.8 |  | P62829 | 38 | 4 | 4 | 40904229.25 |
| P13647 | 40 | 24 | 7 | 679415195.5 |  | F8VQE1 | 12 | 7 | 7 | 11503473.88 |
| Q15149 | 8 | 37 | 36 | 73417852.63 |  | Q2TSD0 | 14 | 5 | 5 | 54423993.88 |
| P11142 | 28 | 15 | 12 | 217345106 |  | P09382 | 32 | 3 | 3 | 30339734.63 |
| B2R853 | 40 | 24 | 1 | 361802304.6 |  | A4ZU86 | 19 | 4 | 4 | 47863944.25 |
| P04259 | 37 | 23 | 1 | 22799914 |  | P52272 | 9 | 7 | 3 | 28015448 |
| P05787 | 38 | 19 | 13 | 321846262.3 |  | Q5TCU3 | 18 | 5 | 2 | 6286923.5 |
| A0A096LPJ3 | 27 | 11 | 1 | 615117462.5 |  | P39019 | 35 | 6 | 6 | 193759175.5 |
| Q13098 | 27 | 11 | 1 | 209214288 |  | B7Z4F6 | 8 | 5 | 5 | 29916671.38 |
| B4DL32 | 57 | 15 | 1 |  |  | P06733 | 13 | 5 | 5 | 10548196.38 |
| Q9UBW8 | 52 | 12 | 12 | 331373428.1 |  | P62280 | 28 | 6 | 6 | 56349553.25 |
| P11021 | 28 | 17 | 15 | 268324381.5 |  | B7Z7S9 | 5 | 6 | 6 | 8192426.875 |
| Q86YZ3 | 13 | 10 | 10 | 49294787.94 |  | A0A2R8YFE2 | 51 | 3 | 1 | 2388275.75 |
| B2RBS8 | 17 | 12 | 12 | 3878233452 |  | P01040 | 53 | 5 | 5 | 74658387.13 |
| M0R0F0 | 41 | 8 | 8 | 280911910.9 |  | P68871 | 27 | 3 | 1 | 15931570 |
| Q04695 | 33 | 14 | 7 | 77142255.25 |  | Q6KB66 | 10 | 4 | 4 | 12398882.5 |
| A8MX94 | 45 | 6 | 6 | 140735111.4 |  | A0A087WYB4 | 19 | 5 | 5 | 17784974.88 |
| A8K3K1 | 25 | 8 | 1 | 4574109.5 |  | Q06830 | 28 | 5 | 4 | 32035653.25 |
| Q96KG9 | 15 | 9 | 9 | 65800525.56 |  | B2RBV7 | 3 | 2 | 2 | 9320556.125 |
| Q16531 | 15 | 15 | 15 | 82868958.5 |  | Q15323 | 12 | 5 | 3 | 13108497.94 |
| E9PGT6 | 44 | 5 | 5 | 294556621.5 |  | Q5D862 | 2 | 4 | 4 | 34692829.88 |
| P08727 | 35 | 14 | 8 | 30306885.69 |  | P04075 | 18 | 7 | 7 | 896665551.5 |
| Q02413 | 11 | 10 | 10 | 31981073.25 |  | Q9HB00 | 6 | 4 | 4 | 15742395.13 |
| Q16195 | 47 | 9 | 1 | 3417157 |  | H0Y449 | 11 | 2 | 2 | 6877568.5 |
| P35580 | 5 | 8 | 2 | 3466121 |  | B4DN72 | 10 | 7 | 1 | 5499751 |
| A0A5E4 | 29 | 5 | 1 | 37741128.75 |  | F8W1S1 | 7 | 5 | 1 |  |
| S6BGD6 | 29 | 5 | 1 | 1559973 |  | Q96T67 | 5 | 3 | 3 | 9877042.063 |
| A0A0G2JIW1 | 19 | 10 | 8 | 27269561.31 |  | Q14525 | 12 | 5 | 3 | 2990283.188 |
| Q8N6N5 | 18 | 7 | 2 | 19588286.5 |  | Q6PEJ8 | 20 | 4 | 4 | 74620879 |
| Q9Y3Z3 | 14 | 7 | 7 | 29141937.25 |  | J3QTR3 | 32 | 3 | 3 | 204129768.8 |
| O95678 | 17 | 12 | 1 | 5320616.5 |  | A8K0T9 | 14 | 3 | 3 | 10599973.25 |
| Q0KKI6 | 37 | 5 | 3 | 38578588.5 |  | P05141 | 16 | 5 | 2 | 143529000.6 |
| B4DY90 | 17 | 7 | 2 | 55981912.25 |  | S6BGE0 | 15 | 4 | 1 |  |
| P07900 | 12 | 8 | 3 | 45637671 |  | P23258 | 6 | 2 | 2 |  |
| P62269 | 38 | 8 | 8 | 2992006947 |  | A6NMY6 | 12 | 4 | 4 | 31203727.75 |
| P05783 | 18 | 8 | 7 | 102058623.1 |  | P05387 | 50 | 3 | 3 | 28924143.63 |
| F8W1R7 | 54 | 7 | 7 | 82870289.25 |  | B4DUQ1 | 6 | 2 | 2 | 2199735 |
| P08238 | 12 | 8 | 3 | 11489302 |  | A0A5C2GBU8 | 17 | 2 | 2 | 69405960.5 |
| Q6N096 | 17 | 7 | 2 | 19652600485 |  | P67936 | 19 | 5 | 2 | 3768528.125 |
| Q16778 | 21 | 3 | 3 | 102730911 |  | Q9BWU5 | 34 | 3 | 1 | 17666714 |
| A0A5C2G5Q9 | 25 | 2 | 2 | 26312870 |  | Q13835 | 2 | 1 | 1 |  |
| P01023 | 3 | 4 | 2 | 21778907 |  | B2R7T8 | 9 | 2 | 2 | 4775177 |
| P26373 | 16 | 3 | 3 | 11404738.75 |  | F5GXS0 | 1 | 2 | 2 | 3267790.875 |
| F5GY37 | 11 | 3 | 3 | 14106600 |  | A0A4D5RAB7 | 10 | 3 | 3 | 2122845.5 |
| Q9BUF5 | 7 | 3 | 1 |  |  | A0A024R326 | 10 | 1 | 1 | 75409240 |
| A0A1W6S962 | 5 | 4 | 4 | 2913923.75 |  | A0A024R5Z9 | 4 | 2 | 2 | 3651537.75 |
| A0A1X7SBS1 | 3 | 2 | 2 | 2144209.5 |  | Q8TA90 | 6 | 3 | 3 | 6538193.875 |
| P31151 | 43 | 4 | 4 | 21242706 |  | A0A087WVQ6 | 2 | 3 | 3 | 6476534.375 |
| M0QZM1 | 13 | 5 | 1 |  |  | B4DM82 | 21 | 3 | 3 | 10765752 |
| P30050 | 28 | 4 | 4 | 46087661.5 |  | P48594 | 5 | 2 | 2 | 6313459.5 |
| H0YAR2 | 16 | 5 | 5 | 16999945.88 |  | P62328 | 41 | 2 | 1 | 32558114 |
| E9PG15 | 17 | 2 | 2 | 5462335.75 |  | S6AWD6 | 19 | 2 | 1 |  |
| A0A024RA28 | 11 | 3 | 3 | 5212262.875 |  | A0A0B4J2D5 | 6 | 2 | 2 | 1872603.5 |
| P25398 | 17 | 1 | 1 |  |  | Q16698 | 6 | 2 | 2 | 11383717 |
| P12236 | 16 | 5 | 2 | 11159194.5 |  | P25705 | 4 | 2 | 2 | 6165135.25 |
| P47929 | 18 | 2 | 2 | 5287246 |  | P80297 | 33 | 2 | 2 | 9238735.5 |
| A0A5C2GP33 | 13 | 1 | 1 | 20712659.25 |  | A0A5C2G1B5 | 18 | 2 | 1 | 5844654.5 |
| M0R210 | 35 | 5 | 5 | 45746037.75 |  | P13010 | 2 | 1 | 1 | 1943293.875 |
| A0A5C2G6V0 | 24 | 2 | 2 | 20629280.5 |  | P36578 | 4 | 2 | 2 | 6557175.375 |
| Q2M2I5 | 6 | 3 | 1 | 873540.5625 |  | Q75MH1 | 13 | 1 | 1 | 3074520.75 |
| B2RDD7 | 5 | 3 | 3 | 13726860 |  | A0A1W6IYL0 | 18 | 2 | 1 | 30435096 |
| Q9H9Q4 | 11 | 2 | 2 | 7478626.75 |  | Q6FG43 | 7 | 2 | 2 | 1167078.875 |
| B4DN41 | 7 | 4 | 4 | 6987963.5 |  | P10599 | 21 | 2 | 2 | 33996378.25 |
| P62805 | 33 | 3 | 3 | 18322977.38 |  | P25786 | 10 | 3 | 3 | 3056803.625 |
| B4DUI5 | 11 | 2 | 2 | 3056067.25 |  | A8K7N0 | 7 | 1 | 1 | 2782153 |
| O00159 | 4 | 4 | 4 | 3728352.875 |  | B2RTS4 | 1 | 1 | 1 | 998747.375 |
| P60866 | 19 | 2 | 2 | 32879142.5 |  | A0A5C2GHE3 | 15 | 1 | 1 | 7123648.5 |
| Q01469 | 21 | 3 | 3 | 41467115 |  | F6RFD5 | 17 | 2 | 2 | 10156557.88 |
| P51991 | 8 | 2 | 2 | 1302145.625 |  | P07814 | 1 | 2 | 2 | 1384741.25 |
| P14923 | 4 | 3 | 3 | 3471086.5 |  | Q1RMG2 | 8 | 2 | 2 |  |
| Q8N720 | 7 | 3 | 3 | 5276709.375 |  | O14950 | 13 | 2 | 2 | 3920013 |
|  |  |  |  |  |  | B2R6K4 | 6 | 2 | 1 | 10583079 |
| P05388 | 15 | 4 | 4 | 16516342.63 |  | Q9H4K7 | 7 | 2 | 2 |  |
| A8MXP9 | 4 | 4 | 4 | 10619074.75 |  | B4DF70 | 10 | 2 | 1 | 3874777.25 |
| A0A5C2GTI8 | 15 | 1 | 1 | 16535754 |  | B4DI38 | 4 | 2 | 2 | 4042107.125 |
| A0A6Q8PFJ0 | 5 | 3 | 3 | 6499326.25 |  | P05109 | 19 | 2 | 2 | 8872517 |
| P18124 | 12 | 3 | 3 | 20811246 |  | E9PFG7 | 2 | 2 | 2 | 3834606.875 |
| P41219 | 6 | 4 | 1 | 4188327.75 |  | K7EMH1 | 27 | 2 | 2 | 1075641.375 |
| B2R8Z8 | 6 | 4 | 4 | 9717689.125 |  | A0A5C2GEZ9 | 11 | 1 | 1 |  |
| P27708 | 1 | 2 | 2 | 4609368.125 |  | J3QQM1 | 5 | 1 | 1 | 2004534.375 |
| B4DN59 | 9 | 3 | 3 | 5064847.25 |  | P63104 | 6 | 1 | 1 | 3802108 |
| C9JRZ6 | 12 | 3 | 3 | 5168750.5 |  | A8K4W0 | 9 | 3 | 3 | 6304731.25 |
| A0A5C2G781 | 15 | 1 | 1 | 106290875 |  | P39023 | 5 | 2 | 2 | 10398657.5 |
| A0A2R8Y811 | 16 | 2 | 2 | 23987803.5 |  | Q59EJ0 | 6 | 2 | 2 | 3548795.75 |
| P02765 | 5 | 3 | 3 | 11608548 |  | I3L3H2 | 6 | 2 | 2 | 1594228.875 |
| A8K6K7 | 4 | 3 | 3 | 6159983.75 |  | Q5T7V8 | 3 | 1 | 1 | 1510366.625 |
| B2R950 | 2 | 3 | 1 | 3282673.25 |  | P35998 | 5 | 2 | 2 | 1629015.5 |
| B7ZKQ8 | 4 | 2 | 2 | 6198481 |  | P19474 | 5 | 2 | 2 | 6258347 |
|  |  |  |  |  |  | Q99729 | 4 | 1 | 1 |  |
| E5RJR5 | 17 | 3 | 3 | 12322767.88 |  | A0A140T907 | 22 | 1 | 1 | 1404922.875 |
| P02656 | 16 | 1 | 1 |  |  | A0A5C2GMX3 | 11 | 1 | 1 | 2130700 |
| B4DWK8 | 7 | 3 | 3 | 7810964.75 |  | P22532 | 31 | 2 | 2 | 8879483 |
| P81605 | 20 | 2 | 2 | 132047563.6 |  | Q96LA8 | 4 | 1 | 1 | 2352368.75 |
| A0A5C2GNF1 | 13 | 1 | 1 | 10737538.38 |  | O00425 | 2 | 1 | 1 | 1419995.5 |
| P16403 | 17 | 3 | 3 | 4759838.75 |  | P62913 | 12 | 2 | 2 | 7998187.25 |
| P08729 | 6 | 4 | 1 | 3327974.75 |  | H0YAF8 | 10 | 2 | 2 | 2988859.5 |
| B0AZN7 | 4 | 3 | 3 | 3934993.75 |  | Q9BQA1 | 7 | 2 | 2 | 5156995.5 |
| C9JXA5 | 9 | 2 | 1 |  |  | Q08188 | 3 | 2 | 2 |  |

a) The unique identifier for a protein in the FASTA database;

b) Coverage%: The percentage of the protein’s amino acid sequence covered by identified peptides;

c) Unique Peptide number: The number of peptides unique to this protein, used for quantification;

d) Peptide number: Total number of peptides identified for the protein, used for qualitative analysis;

e) The relative expression level of the protein, derived from mass spectrometry signal intensity.

**Supplementary Table 2. Clinicopathological features of PDAC patients.**

|  | CSN6 expression | | |  | NPM1 expression | | | | |
| --- | --- | --- | --- | --- | --- | --- | --- | --- | --- |
| Variable | Low | High | *p* value ^a^ |  | Low | | High | | *p* value ^a)^ |
| Gender |  |  | 0.983 |  |  |  | | 0.073 | |
| Male | 38 | 7 |  |  | 32 | 13 | |  | |
| Female | 33 | 6 |  |  | 34 | 5 | |  | |
| Histological grade |  |  | 0.232 |  |  |  | | 0.434 | |
| G1 | 16 | 4 |  |  | 17 | 3 | |  | |
| G2 | 44 | 5 |  |  | 39 | 10 | |  | |
| G3 | 11 | 4 |  |  | 10 | 5 | |  | |
| pT status |  |  | 0.304 |  |  |  | | 0.527 | |
| T1 | 20 | 5 |  |  | 21 | 4 | |  | |
| T2 | 32 | 3 |  |  | 28 | 7 | |  | |
| T3 | 19 | 5 |  |  | 17 | 7 | |  | |
| pN status |  |  | 0.728 |  |  |  | | 0.145 | |
| N0 | 40 | 8 |  |  | 35 | 13 | |  | |
| N1 | 31 | 5 |  |  | 31 | 5 | |  | |
| pM status |  |  | 0.578 |  |  |  | | 0.580 | |
| M0 | 67 | 12 |  |  | 61 | 18 | |  | |
| M1 | 4 | 1 |  |  | 5 | 0 | |  | |
| Clinical stage |  |  | 0.802 |  |  |  | | 0.753 | |
| I | 32 | 5 |  |  | 29 | 8 | |  | |
| II | 35 | 7 |  |  | 32 | 10 | |  | |
| IV | 4 | 1 |  |  | 5 | 0 | |  | |

a) *p* values were calculated in SPSS16.0 using a Pearson Chi-Square Test (Fisher's Exact Test was used when >20% cells have expected count less than 5). *p* values <0.05 were considered to indicate statistical significance.

**Supplemental Table 3. Targeting sequences of shRNAs**

| sh RNAs | Sequence |
| --- | --- |
| sh SC | CTTCTAACACCGGAGGTCTT |
| sh CSN6-1 | CTTGAGAGAAACCGCTGTCAT |
| sh CSN6-2 | CAGTTTGTGAACAAGTTCAAT |
| sh NPM1-1 | GCAAAGGATGAGTTGCACATT |
| sh NPM1-2 | GCCAAGAATGTGTTGTCCAAA |
| sh DCAF1-1 | CCTCCCATTCTTCTGCCTTTA |
| sh DCAF1-2 | CGAGAAACTGAGTCAAATGAA |
| sh CUL4A | GCAGAACTGATCGCAAAGCAT |
| sh CDA | GCCAGTGACATGCAAGATGAT |

**Supplemental Table 4. Primers for qPCR**

| Species | Gene symbol | 5’-3’ | |
| --- | --- | --- | --- |
| Human | ACTB | Forward | CATGTACGTTGCTATCCAGGC |
|  |  | Reverse | CTCCTTAATGTCACGCACGAT |
|  | CSN6 | Forward | TCATCGAGAGCCCCCTCTTT |
|  |  | Reverse | CCAATGCGTTCCGCTTCCT |
|  | pre-rRNA | Forward | TGTCAGGCGTTCTCGTCTC |
|  |  | Reverse | AGCACGACGTCACCACATC |
|  | 5.8-rRNA | Forward | AGCGCTAGCTGCGAGAATTA |
|  |  | Reverse | GACGCTCAGACAGGCGTAG |
|  | 18-rRNA | Forward | CTACTTGGATAACTGTGGTA |
|  |  | Reverse | CGAGGTTATCTAGAGTCAC |
|  | 28-rRNA | Forward | AGAGGTAAACGGGTGGGGTC |
|  |  | Reverse | GGGGTCGGGAGGAACGG |
|  | NPM1 | Forward | GGAGGTGGTAGCAAGGTTCC |
|  |  | Reverse | TTCACTGGCGCTTTTTCTTCA |
|  | DCAF1 | Forward | GGAGGGAATTGTCGAGAATCTTT |
|  |  | Reverse | GTTGGCAGCAATGTCTTGATTT |
|  | CDA | Forward | AAGTCAGCCTACTGCCCCTAC |
|  |  | Reverse | GATAGCGGTCCGTTCAGCAC |
|  | RRM1 | Forward | GCCAGGATCGCTGTCTCTAAC |
|  |  | Reverse | GAGAGTGTTTGCCATTATGTGGA |
|  | RRM2 | Forward | CACGGAGCCGAAAACTAAAGC |
|  |  | Reverse | TCTGCCTTCTTATACATCTGCCA |
|  | H42.1 | Forward | GCTTCTCGACTCACGGTTTC |
|  |  | Reverse | CCGAGAGCACGATCTCAAA |
|  | H42.9 | Forward | CCCGGGGGAGGTATATCTTT |
|  |  | Reverse | CCAACCTCTCCGACGACA |
|  | H1 | Forward | GGCGGTTTGAGTGAGACGAGA |
|  |  | Reverse | ACGTGCGCTCACCGAGAGCAG |
|  | H4 | Forward | CGACGACCCATTCGAACGTCT |
|  |  | Reverse | CTCTCCGGAATCGAACCCTGA |
|  | H8 | Forward | AGTCGGGTTGCTTGGGAATGC |
|  |  | Reverse | CCCTTACGGTACTTGTTGACT |
|  | H13 | Forward | ACCTGGCGCTAAACCATTCGT |
|  |  | Reverse | GGACAAACCCTTGTGTCGAGG |
| Mouse | ACTB | Forward | TAGGCACCAGGGTGTGATGG |
|  |  | Reverse | CATGGCTGGGGTGTTGAAGG |
|  | CSN6 | Forward | AGCACTGACAAGTTCAAGACA |
|  |  | Reverse | GTGATGGTGCCGAGGTAG |
|  | pre-rRNA | Forward | AAATAAGGTGGCCCTCAACC |
|  |  | Reverse | CGTGCCGGTATTTAGCCTTA |
|  | 5.8-rRNA | Forward | GACTCTTAGCGGTGGATCACT |
|  |  | Reverse | CGTTCTTCATCGACGCACGA |
|  | 18-rRNA | Forward | GTAACCCGTTGAACCCCATT |
|  |  | Reverse | CCATCCAATCGGTAGTAGCG |
|  | 28-rRNA | Forward | GGTTGAGGGCCACCTTATTT |
|  |  | Reverse | GAAGAAAGACCGGGAAGAGAAA |
